# Integrative transcriptomic and computational analysis associates Ms4a6c+ macrophages with fibrosis in radiation proctitis and computationally nominates MOTS-c as a candidate modulator

**DOI:** 10.3389/fimmu.2026.1907084

**Published:** 2026-10-14

**Authors:** Peng Wang, Wei Tao, Hong Wen, Kai Liu, Yuanyuan Fu, Yanze Kan, Teming Li, Zhiqiang Zhang, Weidong Xiao

**Affiliations:** Key Laboratory for Precision Digestive Surgery and Intestinal Homeostasis, Department of General Surgery, The Second Affiliated Hospital of Army Medical University, Chongqing, China

**Keywords:** Cebpb, chronic radiation proctitis, cross-disease validation, fibrosis, molecular dynamics simulations, Ms4a6c, Thbs1-Cd47

## Abstract

**Background:**

Chronic radiation proctitis (CRP) represents a significant clinical challenge characterized by irreversible fibrosis and a lack of specific pharmacological interventions. This study aimed to elucidate the cellular mechanisms underlying impaired tissue repair to identify novel therapeutic targets.

**Methods:**

We employed an integrative framework combining machine learning with bulk and high-resolution single-cell transcriptomic profiling in a murine CRP model. Computational mechanistic analyses included regulon inference, cell-cell communication analysis, and single-cell virtual knockout simulation. The pro-fibrotic association of the identified macrophage subset was further examined using independent single-cell datasets from liver cirrhosis and idiopathic pulmonary fibrosis. Furthermore, structure-based virtual screening and molecular dynamics simulations were conducted to identify potential pharmacological modulators.

**Results:**

We identified Ms4a6c as a key candidate gene exclusively expressed in a distinct, hybrid-activation macrophage subpopulation associated with pro-fibrotic transcriptional programs. These macrophages showed predicted associations with fibrosis via Thbs1-Cd47 paracrine signaling interactions with fibroblasts, and SCENIC regulon analysis identified Cebpb as a candidate upstream transcriptional regulator. Cross-disease analysis in liver cirrhosis and idiopathic pulmonary fibrosis single-cell datasets indicated that Ms4a6c-high macrophage subsets are consistently associated with elevated epithelial-mesenchymal transition and TGF-β signaling activity, supporting a potentially conserved pro-fibrotic role across organ systems. Furthermore, single-cell virtual knockout simulation predicted that the computational depletion of Ms4a6c may reverse pro-fibrotic transcriptomic signatures. Additionally, molecular docking and dynamics simulations identified the mitochondrial-derived peptide MOTS-c as a top-ranking candidate predicted to target Ms4a6c.

**Conclusions:**

This study highlights the potential pathogenic involvement of Ms4a6c+ macrophages and identifies the Cebpb-Ms4a6c-Thbs1-Cd47 axis as a candidate pro-fibrotic signaling cascade that may be conserved across multiple organ fibrosis models. We propose MOTS-c as a potential pharmacological agent for mitigating radiation-induced intestinal fibrosis, pending experimental validation.

## Introduction

Pelvic radiotherapy is a pivotal treatment for colorectal, gynecological, prostate, and bladder cancers, and is indicated in approximately half of all patients with cancer (1). Radiation-induced rectal injury remains a substantial clinical burden in China, where dedicated management guidelines have recently been issued (2). However, despite precision techniques like IMRT, chronic radiation proctitis (CRP) remains a debilitating complication, affecting 5–20% of survivors, with 2–5% suffering severe sequelae (3–5). Typically emerging 3–6 months post-treatment, CRP progresses from symptoms like bleeding and tenesmus to severe fibrotic strictures and obstruction, significantly burdening healthcare systems (6, 7).

Pathophysiologically, CRP evolves from acute radiation-induced DNA damage and inflammation to chronic maladaptive remodeling, characterized by aberrant extracellular matrix deposition and myofibroblast differentiation (8, 9). Current clinical management remains largely empirical and palliative, relying on topical agents, hyperbaric oxygen, or endoscopic coagulation, with surgery reserved for refractory cases (10). Crucially, no FDA-approved pharmacological therapies exist to effectively reverse radiation-induced intestinal fibrosis, a deficit stemming from an incomplete understanding of the underlying cellular and molecular mechanisms.

Macrophages are established regulators of fibrosis in organs such as the kidney, liver, lung, and heart (11–14). However, the specific macrophage subsets driving radiation-induced intestinal fibrosis remain unidentified. While the traditional M1/M2 binary classification is insufficient, recent single-cell RNA sequencing (scRNA-seq) studies have revealed distinct pro-fibrotic macrophage populations in various tissues (15, 16). Yet, the identity and regulatory dynamics of these populations within the irradiated intestine remain largely uncharacterized.

Our investigation highlights the MS4A gene family, particularly Ms4a6c, a membrane-spanning protein expressed in hematopoietic cells (17). Although Ms4a6c marks alternatively activated macrophages in some contexts, its function in radiation-induced fibrosis is unexplored (18, 19). Elucidating the transcriptional regulation of these macrophages could reveal novel therapeutic targets. Furthermore, to understand macrophage-stromal communication, we examined Thrombospondin-1 (Thbs1), a matricellular protein known to activate TGF-β (20). While Thbs1 promotes fibroblast activation via CD47 in other fibrotic diseases (21, 22), its specific role in mediating macrophage-fibroblast crosstalk in CRP remains to be defined.

In this study, we employed an integrative transcriptomic and computational approach, combining bulk RNA sequencing with high-resolution scRNA-seq (>62,000 cells) to map the cellular landscape of CRP. Using machine learning algorithms (LASSO, SVM-RFE) and trajectory analysis, we identified Ms4a6c as a candidate marker of a distinct pro-fibrotic macrophage subset. We further examined the pro-fibrotic association of Ms4a6c+ macrophages using independent single-cell RNA sequencing datasets from liver cirrhosis (GSE136103) and idiopathic pulmonary fibrosis (GSE135893) and identified Cebpb as a candidate upstream transcriptional regulator via regulon inference. Finally, structure-based virtual screening identified MOTS-c as a potential therapeutic modulator of Ms4a6c, providing a foundation for future *in vitro* and *in vivo* functional validation and drug development.

## Materials and methods

### Ethical statement and patient consent

This study adhered to the Declaration of Helsinki and international animal care guidelines. Approvals were granted by the Laboratory Animal Welfare and Ethics Committee (AMUWEC20235020) and the Ethics Committee of the Second Affiliated Hospital of Army Medical University (2025-Research-251-01). Written informed consent was obtained from all patients providing paired rectal tissues (irradiated peri-tumor vs. non-irradiated distal normal) during surgical resection.

### Experimental animals and CRP model

Sex-balanced C57BL/6J mice (6–8 weeks; Hunan SJA Laboratory Animal Co.) were housed under SPF conditions. To model CRP, a custom 3D-printed restraint with 4-mm lead shielding was utilized for precise pelvic irradiation. Prior to irradiation, mice were anesthetized by intraperitoneal injection of 2,2,2-tribromoethanol (Cat# T48402, Sigma-Aldrich, St. Louis, MO, USA) at approximately 250 mg/kg. The anesthetic working solution was freshly prepared by dissolving 0.25 g of 2,2,2-tribromoethanol in 1 mL of absolute ethanol and then diluting with sterile physiological saline to a final volume of 10 mL (working concentration, 25 mg/mL); each mouse received approximately 200 μL of this solution by intraperitoneal injection. The anesthetized mice received a single 25 Gy fraction (10×15 mm aperture, 5 mm from anus) via a Raycision Medical Technology Co., Ltd unit (SHAPP100) (225 kV, 12.5 mA, 1.75 Gy/min). Sham controls underwent identical handling without radiation. At the experimental endpoint (week 8), a timepoint corresponding to the established chronic fibrotic phase in localized pelvic-irradiation RP models (23, 24), all mice were euthanized by cervical dislocation, and rectal tissues were harvested for downstream analyses.

### Clinical and histological assessment

Body weight and Disease Activity Index (DAI; **Supplementary Table 1**) were monitored weekly (25). At week 8, mucosal injury was assessed via high-resolution colonoscopy (ENDOQ, KARL STORZ) using a validated scoring system (**Supplementary Table 2**) (26). Tissues were FFPE-processed and stained with H&E, Masson’s trichrome, and Sirius Red. Fibrosis was scored by blinded pathologists (**Supplementary Table 3**) (27). Multiplex immunofluorescence was performed using the Opal kit (Akoya Biosciences) with antibodies against F4/80 (Catalog: sc-71085, Santa Cruz, dilution: 1:200), Ms4a6c (Catalog: ER1913-19, HuaBio, dilution: 1:150), PDGFR-α (Catalog: sc-398206, Santa Cruz, dilution: 1:200), CD47 (Catalog: DF6649, Affinity, dilution: 1:200), and THBS1 (Catalog: HA721916, HuaBio, dilution: 1:200). For human tissue staining, this antibody (ER1913-19; an anti-MS4A6A rabbit polyclonal with validated human, mouse and rat reactivity) detects the human MS4A6A ortholog. Ms4a6c+F4/80+ cells were counted and expressed as a percentage of F4/80+ macrophages (3 random fields per sample averaged, blinded; n=5 per group).

### Bulk RNA sequencing and machine learning

Total RNA (TRIzol; Solarbio) meeting quality standards (RIN > 7.0) was sequenced on an Illumina NovaSeq 6000 (PE150). Data were processed via fastp, HISAT2, and featureCounts. DEGs were identified using DESeq2 (|log2FC> 0.585, adj. P < 0.05). Enrichment analyses utilized GSEA (fgsea) and clusterProfiler. As an exploratory feature-prioritization step, LASSO regression (glmnet) and SVM-RFE (e1071) were applied to the bulk RNA-seq data (n=5 per group) and their selected features intersected to obtain a short candidate list for downstream single-cell analysis.

### Single-cell RNA sequencing analysis

Single-cell suspensions were processed (10x Genomics v2) and sequenced at NovelBio Bio-Pharm Technology Co., Ltd. (Shanghai, China). High-quality cells (62,913; filtered for doublets/mitochondrial content) were analyzed using Seurat v4.3.0 (normalization, CCA integration, Leiden clustering). Macrophages were split at the median Ms4a6c expression into Ms4a6c+ (high) and Ms4a6c− (low) subsets (n = 2,028 cells each). Advanced analyses included CellChat v1.4.0 (intercellular communication), Monocle v2.34.0/CytoTRACE (trajectory), and SCENIC v1.3.1 (regulon inference). In silico perturbation of Ms4a6c was simulated using scTenifoldKnk (28), based on a gene regulatory network constructed from the validated macrophage subset (after doublet removal with DoubletFinder and standard quality-control filtering), to identify differentially regulated genes and associated pathways.

### Cross-disease validation using external single-cell datasets

To assess the cross-disease relevance of Ms4a6c+ macrophages, we analyzed two independent publicly available single-cell RNA sequencing datasets: (1) liver cirrhosis (LC; GSE136103) and (2) idiopathic pulmonary fibrosis (IPF; GSE135893). For both datasets, macrophage populations were extracted based on canonical markers (CD68, CSF1R, CD163, MARCO; **Supplementary Figure S4**) and sub-clustered using Seurat v4.3.0. An Ms4a6c-like scoring metric was computed using the AddModuleScore function based on an Ms4a6c+ macrophage gene signature derived from our CRP model. This signature comprised the top genes significantly up-regulated in Ms4a6c-high versus Ms4a6c-low macrophages in the murine CRP dataset (FindMarkers, adjusted P < 0.05, ranked by log2 fold-change), converted to human orthologs (Ms4a6c and MS4A6A are annotated orthologs; because automated one-to-one mapping is ambiguous for this rodent-expanded subfamily, this correspondence was mapped manually and confirmed by sequence and structural alignment, **Supplementary Figures S6A, B**), yielding a 52-gene human signature (**Supplementary Table 6**). To exclude circularity between the defining signature and the scored pathways, the signature was intersected with the EMT and TGF-β gene sets, sharing no genes with the former and a single gene (THBS1, a core axis gene) with the latter. Epithelial-mesenchymal transition (EMT) and TGF-β signaling activity scores were calculated using the AUCell algorithm. Wilcoxon rank-sum tests were used to compare pathway activity between Ms4a6c-high and Ms4a6c-low macrophage subsets. To account for the non-independence of cells from the same sample (pseudoreplication), these comparisons were additionally performed at the sample level. Within each dataset, Ms4a6c-high and Ms4a6c-low macrophages were defined as the top and bottom 20% of the Ms4a6c-like score computed within each individual sample (for the CRP model, the annotated Ms4a6c-high and Ms4a6c-low subsets were used), the EMT and TGF-β AUCell scores were averaged per animal or patient, and the paired per-sample values were compared using a paired t test.

### Molecular validation (qRT-PCR and Western blot)

qRT-PCR was conducted using the PrimeScript RT Kit (Solarbio) and QuantStudio 5 system, and relative expression was determined using the comparative Ct method. For Western blot, lysates (RIPA/BCA) were resolved by SDS-PAGE and probed with primary antibodies against Ms4a6c (Catalog: ER1913-19, HuaBio, dilution: 1:500), Col6a2 (Catalog: ER63079, HuaBio, dilution: 1:500), and Ncf1 (Catalog: ER64501, HuaBio, dilution: 1:500), as well as β-actin (Catalog: AF2811, Beyotime, dilution: 1:1000). Signals were visualized using ECL reagent (Catalog: P0018S, Beyotime). The qRT-PCR primer sequences (5’→3’) were as follows: Ms4a6c, forward TCAAAGTGATAGTGGCAATCCAG, reverse CCCTTCTCTGTCTTCCCCCAT; Col6a2, forward CCAGTCAACGTGTATTTCGTGT, reverse TAGGCTCTTAGTGAAGGAGGC; Ncf1, forward ACACCTTCATTCGCCATATTGC, reverse CCTGCCACTTAACCAGGAACA.

### Virtual screening and MD simulations

The three-dimensional (3D) protein structure of murine Ms4a6c was retrieved from the AlphaFold Protein Structure Database (Accession ID: AF-Q99N08-2-F1). This AI-predicted high-confidence structural model served as the biological receptor for subsequent computational investigations. The structure was pre-processed using the ‘Prepare Protein’ module within Discovery Studio 2021 to assign partial charges, optimize hydrogen bond networks, and resolve potential steric clashes. Initial high-throughput virtual screening was conducted against a combined panel of 11 MedChemExpress (MCE) compound libraries totaling 58,581 compound entries (not de-duplicated across libraries) using the LibDock algorithm, which utilized a grid-based approach for ligand alignment. The resulting top-ranking poses were further refined and re-scored using AutoDockTools to ensure optimal binding orientations and energetic compatibility.

To evaluate the stability and binding affinity of the prioritized complexes, 100-ns MD simulations were performed using GROMACS 2020.6 with the CHARMM36 all-atom force field. Each complex was immersed in a cubic water box solvated with TIP3P water molecules and neutralized by adding Na+ or Cl- ions to reach a physiological salt concentration (0.15 M). The systems underwent energy minimization, followed by NVT and NPT equilibration to achieve temperature (300 K) and pressure (1 bar) stability. Production runs were executed for 100 ns with a 2-fs time step. The resulting trajectories were comprehensively analyzed to assess structural integrity and thermodynamics, including Root Mean Square Deviation (RMSD), Root Mean Square Fluctuation (RMSF), Radius of Gyration (Rg), hydrogen bonding occupancy, and Gibbs free energy landscapes (FEL) calculated via principal component analysis (PCA).

### Statistical analysis

Data are presented as mean ± SEM. Group comparisons were performed using unpaired t tests (two independent groups) or one-way ANOVA with Tukey’s *post hoc* test (more than two groups) in GraphPad Prism 9.0 and R 4.1.0. For the immunofluorescence quantification of Ms4a6c+ macrophage density, human samples—paired peri-tumor irradiated versus non-irradiated distal normal rectal tissue from the same patient—were compared by paired t test, whereas mouse irradiated versus sham (lead-shielded) groups, comprising independent animals, were compared by unpaired t test. P < 0.05 was considered significant (*P < 0.05, **P < 0.01, ***P < 0.001, ****P < 0.0001).

## Results

### A localized pelvic irradiation mouse model recapitulates key fibrotic and inflammatory features of chronic radiation proctitis

To elucidate mechanisms of radiation-induced intestinal fibrosis, we established a CRP mouse model using custom 3D-printed devices for precise localized pelvic irradiation (25 Gy; **Figure 1A**, **S1A–D**). Over 8 weeks, irradiated mice of both sexes showed progressive pathology, including significant weight loss (**Figure 1B**, **S1E**), elevated disease activity index (**Figure 1C**, **Supplementary S1F**), and symptoms like diarrhea and hematochezia (**Figure 1D**, **S1G**). Week 8 colonoscopy revealed severe mucosal damage (vascular loss, edema, ulceration; **Figure 1E**, **Supplementary S1H–I**) and significantly elevated colonoscopy scores (P < 0.001; **Figure 1F**). Histopathology confirmed extensive fibrosis, showing marked increases in histologic scores, fibrotic area, and collagen deposition (**Figures 1G–J**, **S2A**). Fibrosis was spatially confined to the distal rectum (**Supplementary Figures S2B, C**).

**Figure 1:**
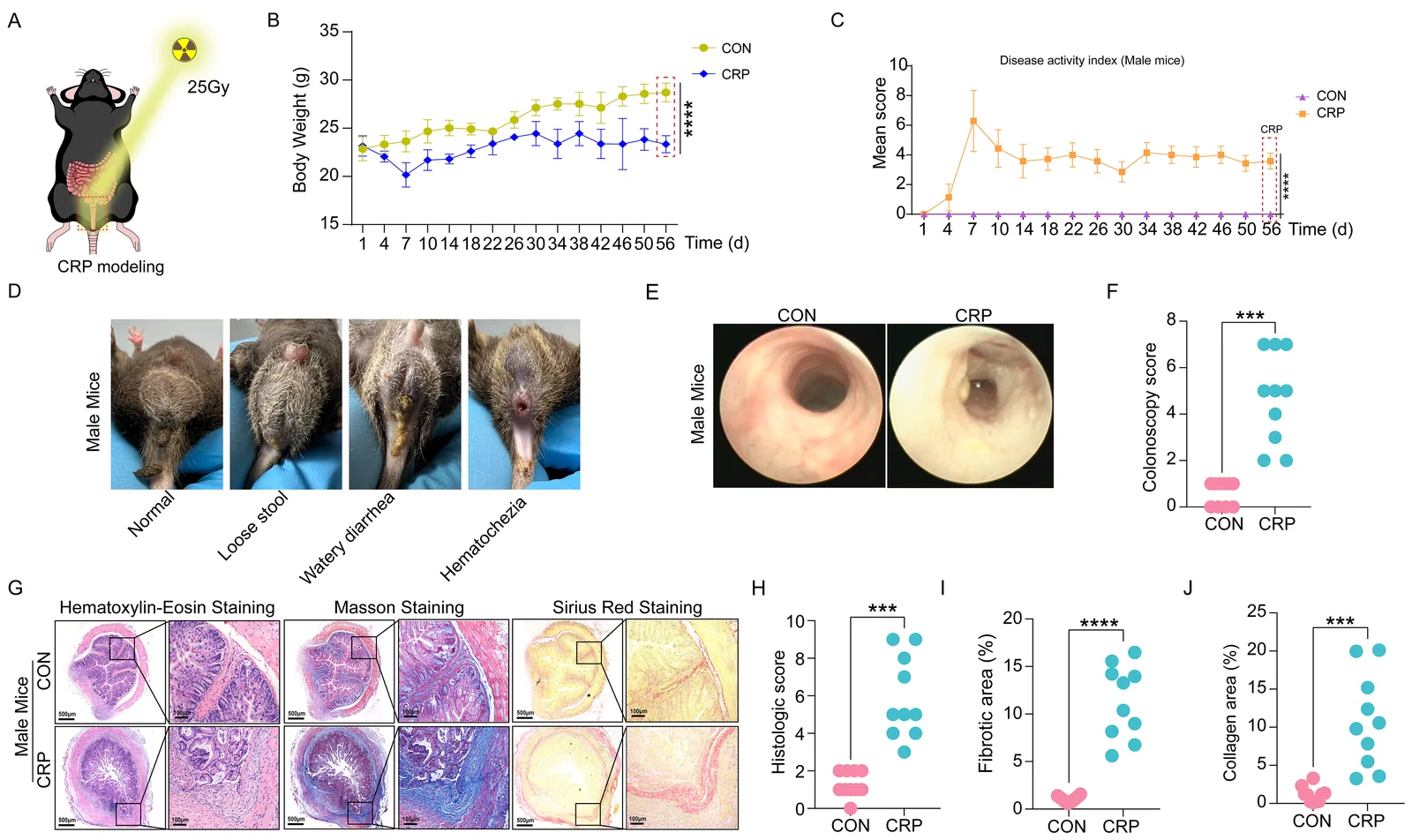
Establishment and characterization of a chronic radiation proctitis mouse model. **(A)** Schematic of localized pelvic irradiation (25 Gy). **(B)** Body weight changes in male mice (n=10/group). **(C)** Longitudinal Disease Activity Index (DAI) scores (n=10/group). **(D)** Representative gross pathology showing diarrhea and hematochezia. **(E)** Representative colonoscopic images at 8 weeks post-irradiation. **(F)** Colonoscopy scores (n=10/group). **(G)** Representative H&E, Masson’s trichrome, and Sirius Red staining. Scale bars: 500 μm (upper), 100 μm (lower). **(H–J)** Histological scoring, fibrotic area, and collagen area quantification (n=10/group). Data are mean ± SEM. ***P<0.001, ****P<0.0001.

### Dual machine learning approaches identify Ms4a6c as a candidate gene for radiation-induced fibrosis

Bulk RNA sequencing of rectal tissues (8 weeks post-irradiation, n=5) revealed distinct group segregation (PC1: 96.5% variance), confirming significant transcriptomic reprogramming (**Figure 2A**). We identified 408 upregulated and 92 downregulated genes (**Figure 2B**), with GSEA showing enrichment in fibrotic hallmarks including ECM organization, inflammation, and EMT (**Figures 2C, D**). To prioritize candidate features, LASSO regression identified six candidates (**Figures 2E, F**), while SVM-RFE selected 40 feature genes as its optimal subset (**Figures 2G, H**). The convergence of these methods yielded five convergent candidate genes: Ms4a6c, Col6a2, Zfp708, Flnc, and Ncf1 (**Figure 2I**). Validation confirmed Ms4a6c and Col6a2 upregulation at transcript (**Figures 2J, K**) and protein levels (**Figures 2L, M**). By contrast, although Ncf1 was one of the five convergent candidate genes, its increase at the protein level did not reach statistical significance (**Figure 2M**), possibly reflecting post-transcriptional regulation. Unlike the structural protein Col6a2, Ms4a6c is an immune-related membrane protein, suggesting a potential regulatory role that warrants further functional investigation.

**Figure 2:**
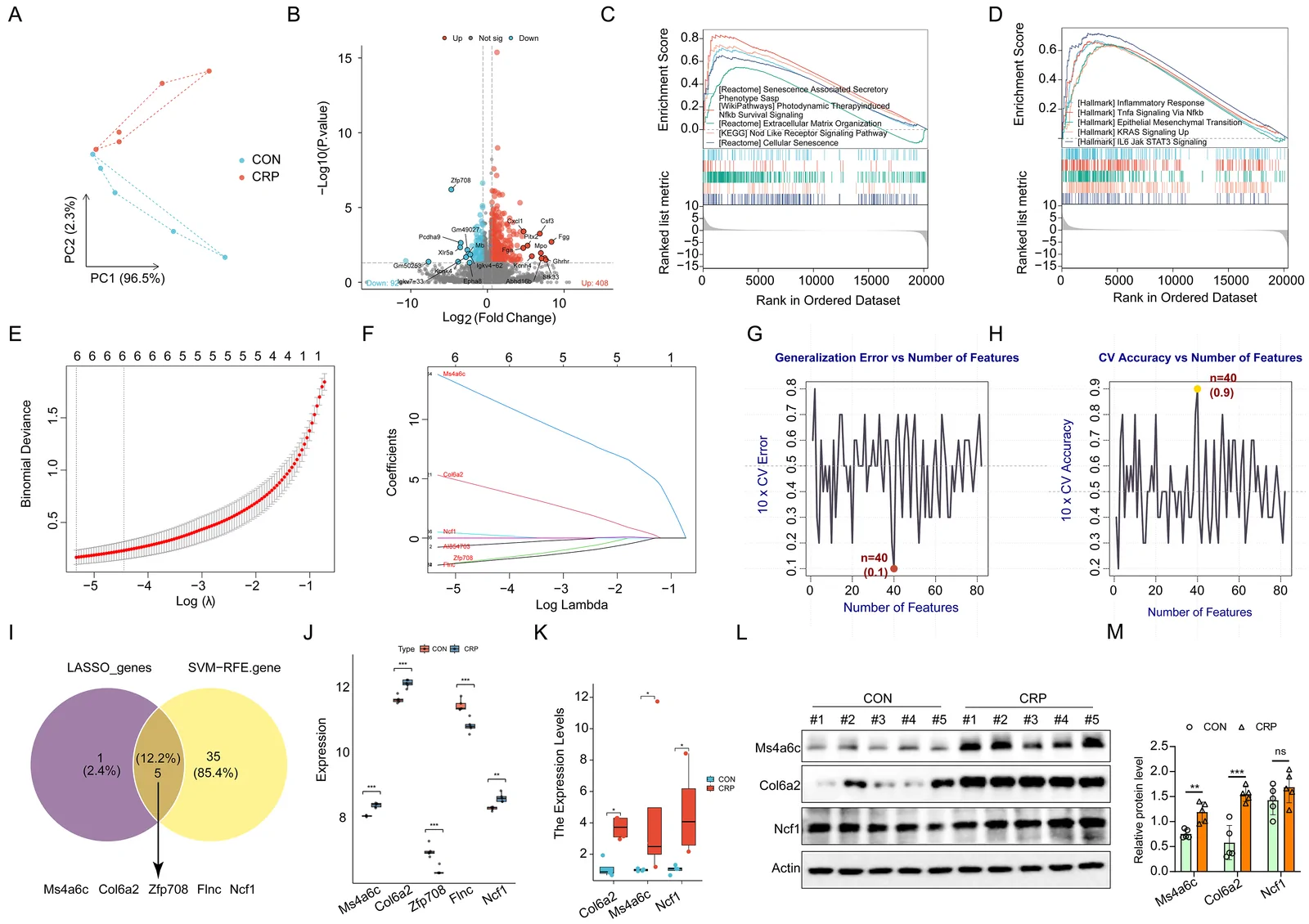
Identification of Ms4a6c as a candidate gene through transcriptomic profiling and machine learning. **(A)** PCA plot of bulk RNA-seq data (n=5/group). **(B)** Volcano plot of differentially expressed genes (DEGs). **(C, D)** GSEA of KEGG pathways and Hallmark gene sets. **(E, F)** LASSO regression with cross-validation. **(G, H)** SVM-RFE feature elimination. **(I)** Venn diagram of the intersection between LASSO and SVM-RFE, identifying five convergent candidate genes. **(J)** RNA-seq expression of candidate genes. **(K)** RT-qPCR validation (n=5/group). **(L, M)** Western blot bands **(L)** and corresponding bar-graph quantification **(M)** of Ms4a6c, Col6a2, and Ncf1, normalized to β-actin (n=5/group). Data are mean ± SEM; *P<0.05, **P<0.01, ***P<0.001; ns, not significant.

### Single-cell profiling identifies Ms4a6c+ macrophages as a transcriptionally distinct subset characterized by dual inflammatory and pro-fibrotic signatures

scRNA-seq analysis of 62,913 high-quality cells (**Supplementary Figures S3A–C**) delineated nine lineages via UMAP (**Figure 3A**), annotated using canonical markers (**Figure 3B**). Detailed feature plots confirmed the identity of structural and immune populations (**Supplementary Figures S3E–M**). CRP tissues exhibited significant immune expansion, notably in macrophages, B cells, and plasma cells, concurrent with epithelial reduction (**Supplementary Figure S3D**). Ms4a6c was exclusively restricted to macrophages (**Figures 3C, D**), supported by co-localization with F4/80 (but not PDGFR-α) in human and mouse tissues (**Figures 3E, F**). Macrophages were stratified into Ms4a6c+ and Ms4a6c− subsets (**Figure 3G**), with the human ortholog MS4A6A enriched in M2 macrophages (**Figure 3H**). Ms4a6c+ macrophages displayed a hybrid activation state, co-enriching TGF-β/M2 and NF-κB markers (**Figures 3I–K**), alongside oxidative phosphorylation, EMT, and angiogenesis signatures (**Figures 3L, M**). Consistent with a pro-fibrotic phenotype, Ms4a6c expression showed a weak but statistically significant positive correlation with EMT and TGF-β signaling scores at single-cell resolution (EMT: R = 0.16, R^2^ = 0.026, P = 3.09 × 10^–19^; TGF-β: R = 0.19, R^2^ = 0.036, P = 3.63 × 10^–31^; **Figures 2N, O**, left), a magnitude expected for single-gene versus pathway-score correlations in sparse single-cell data. At the subset level, Ms4a6c+ macrophages exhibited significantly higher EMT and TGF-β signaling scores than Ms4a6c− macrophages (two-sided Wilcoxon rank-sum test; EMT P = 3.66 × 10^–37^, TGF-β P = 1.56 × 10^–49^; **Figures 3N, O**, right).

**Figure 3:**
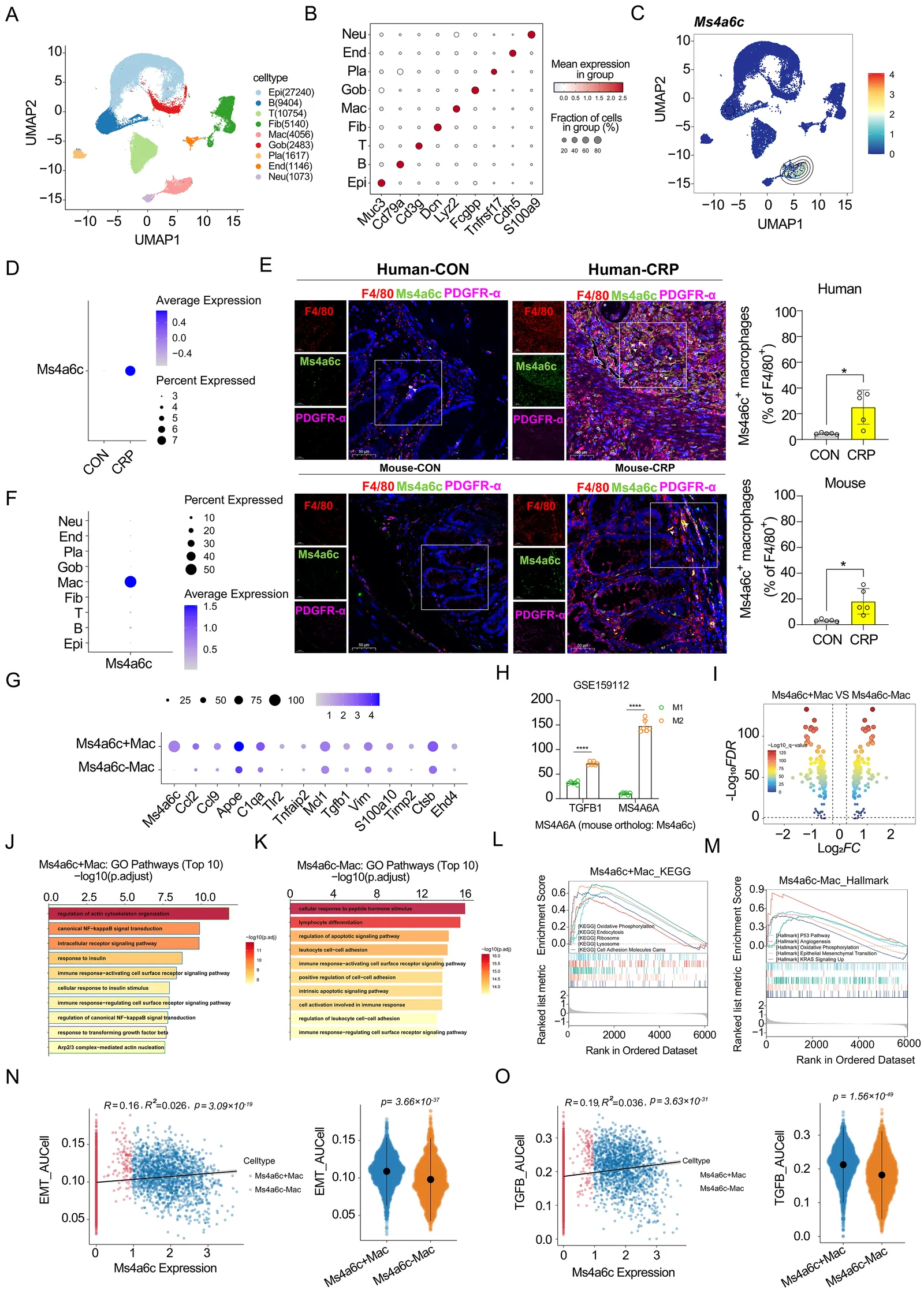
Single-cell transcriptomics identifies Ms4a6c as a macrophage-specific marker associated with pro-fibrotic gene signatures. **(A)** UMAP visualization of 62,913 cells (Control n=4, CRP n=5). **(B)** Dot plot of canonical markers. **(C, D)** UMAP plot and quantification of Ms4a6c expression across tissues. **(E)** Multiplex immunofluorescence of Ms4a6c (green), F4/80 (red), and PDGFR-α (magenta) in human and mouse CRP versus control tissues (DAPI: blue; n=5 CRP and n=5 control per species). Scale bars: 50 μm. Right, quantification of Ms4a6c+ macrophage density (Ms4a6c+ cells as % of F4/80+) in human (upper) and mouse (lower) CON versus CRP. Data are mean ± SEM; *P<0.05 (human: paired t test, P = 0.0268; mouse: unpaired t test, P = 0.014). **(F)** Ms4a6c expression across cell types. **(G)** Ms4a6c heterogeneity in macrophages (Ms4a6c+ macrophages, n =2028 cells; Ms4a6c− macrophages, n = 2028 cells). **(H)** MS4A6A enrichment in M2 macrophages (GSE159112) (M1: n=6, M2: n=6; ****P < 0.0001, unpaired t test). **(I)** DEGs between Ms4a6c+ and Ms4a6c− macrophages. **(J–K)** GO enrichment analysis. **(L, M)** KEGG and Hallmark pathway analyses. **(N, O)** Correlation of Ms4a6c expression with EMT and TGF-β signaling scores (left; Pearson R, R^2^, and P indicated) and comparison between Ms4a6c+ and Ms4a6c− macrophage subsets (right; two-sided Wilcoxon rank-sum test).

### Ms4a6c+ macrophages exhibit enhanced predicted communication with fibroblasts via the Thbs1-Cd47 axis

CellChat analysis predicted that Ms4a6c+ macrophages participate in extensive signaling networks, particularly with fibroblasts (**Figures 4A, B**). The Thbs1-Cd47 axis exhibited the highest communication probability (**Figure 4C**); Ms4a6c+ macrophages co-expressed high levels of Ms4a6c and the ligand Thbs1, while the receptor Cd47 was widely expressed, including on fibroblasts (**Figures 4D–G**). Dotplot showed the upregulation of Thbs1 and Cd47 in CRP tissues (**Figure 4H**). Furthermore, multiplex immunofluorescence showed spatial co-localization of Ms4a6c, Thbs1, and Cd47 within fibrotic niches (**Figure 4I**), providing *in situ* evidence consistent with, but not proof of, the predicted paracrine interaction; direct functional validation remains necessary.

**Figure 4:**
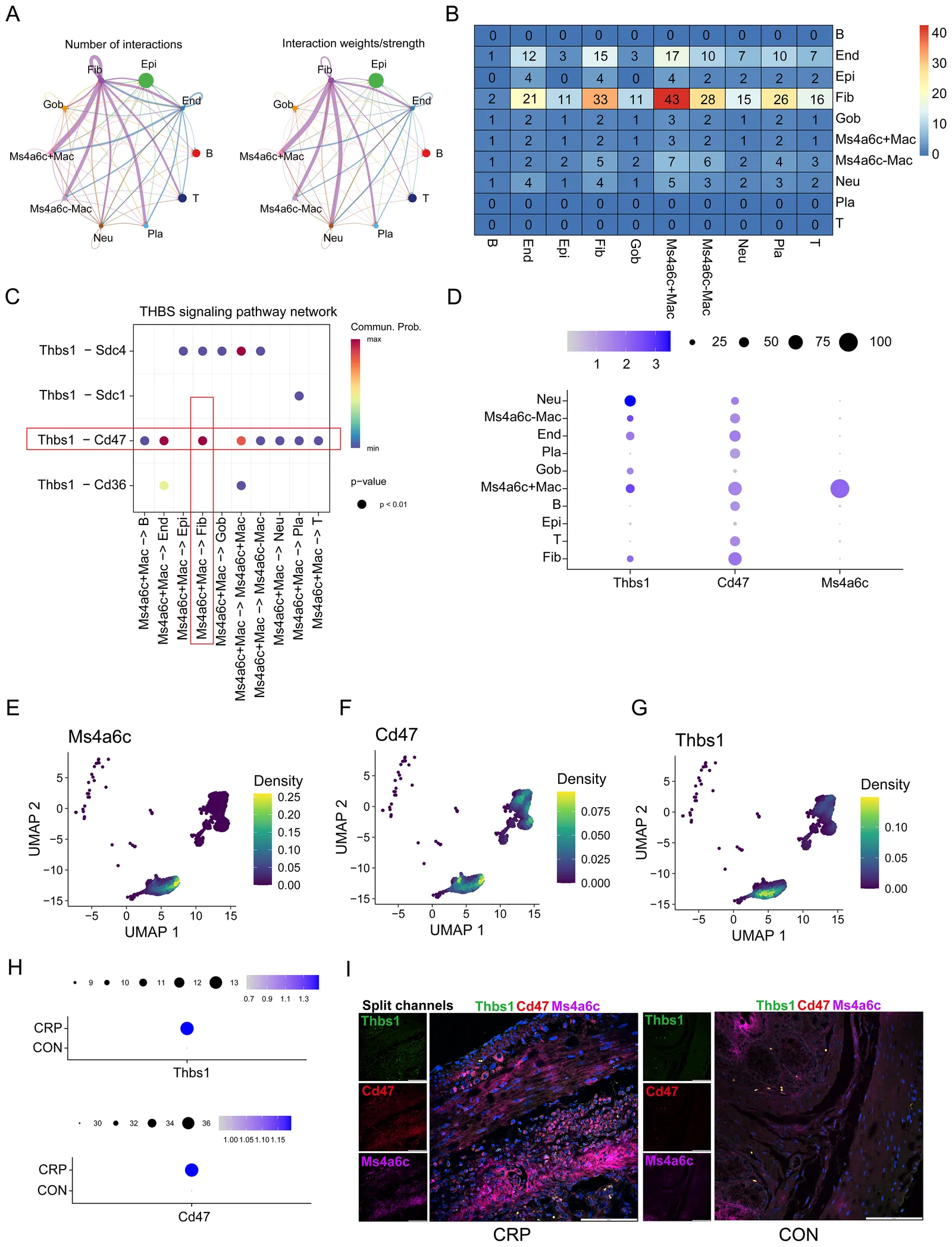
Ms4a6c+ macrophages show predicted communication with fibroblasts through Thbs1-Cd47 signaling. **(A)** CellChat network diagrams illustrate the number and strength of predicted cellular interactions. **(B)** A heatmap depicts the interaction strength between various cell types. **(C)** Communication probabilities within the THBS signaling pathway are presented. **(D)** The expression levels of Ms4a6c, Thbs1, and Cd47 across different cell types are analyzed. **(E–G)** UMAP density plots display the distribution of Ms4a6c+ macrophages and the expression patterns of Cd47 and Thbs1. **(H)** A dot plot visualizes the expression levels and the percentage of cells expressing Thbs1 and Cd47 in CRP versus control groups. **(I)** Multiplex immunofluorescence images show the localization of Ms4a6c (magenta), Thbs1 (green), and Cd47 (red) in both CRP and control groups, with DAPI (blue) staining the nuclei. Scale bars represent 100 μm.

### Cross-disease analysis and regulon inference identify Ms4a6c+ macrophages as candidate conserved pro-fibrotic effectors with Cebpb as a potential upstream regulator

To assess the generalizability of Ms4a6c+ macrophages as pro-fibrotic effectors beyond radiation-induced intestinal fibrosis, we analyzed independent single-cell RNA sequencing datasets from liver cirrhosis (LC; GSE136103) and idiopathic pulmonary fibrosis (IPF; GSE135893). In the LC dataset, macrophage sub-clustering identified seven distinct populations (**Figure 5A**; **Supplementary Table 5**), and an Ms4a6c-like signature score was computed across all sub-clusters (**Figure 5B**). Macrophages with high Ms4a6c-like scores exhibited significantly elevated EMT activity (Wilcoxon P = 3.6e-07; **Figure 5C**) and TGF-β signaling activity (Wilcoxon P = 1.5e-13; **Figure 5D**) compared to the Ms4a6c-low subset (this liver-cirrhosis association was weaker in the per-sample analysis). Similarly, in the IPF dataset, macrophage sub-clustering revealed nine populations (**Figures 5E, F**), and the Ms4a6c-high macrophage subset again showed significantly higher EMT activity (Wilcoxon P < 2e-16; **Figure 5G**) and TGF-β signaling activity (Wilcoxon P < 2e-16; **Figure 5H**) relative to the Ms4a6c-low subset.

**Figure 5:**
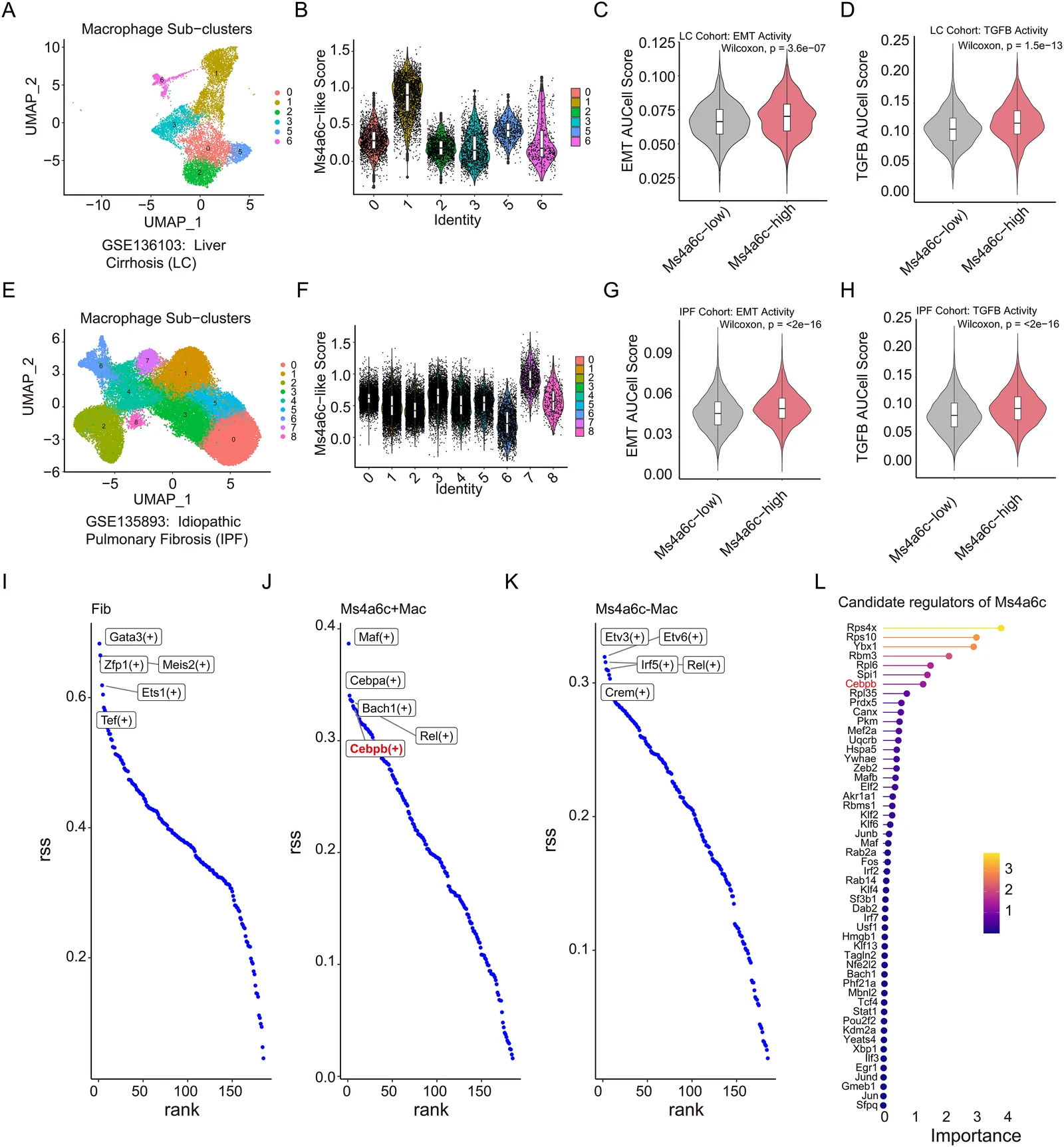
Cross-disease analysis of Ms4a6c+ macrophages and identification of candidate transcriptional regulators. **(A)** UMAP visualization of macrophage sub-clusters in LC. **(B)** Violin plots of Ms4a6c-like signature scores across LC macrophage sub-clusters (sub-cluster 4 was merged into sub-cluster 1 during annotation; six sub-clusters are shown). **(C)** Violin plot comparing EMT activity between Ms4a6c-low and Ms4a6c-high macrophages in the LC cohort. **(D)** Violin plot comparing TGF-β signaling activity between Ms4a6c-low and Ms4a6c-high macrophages in the LC cohort. **(E)** UMAP visualization of macrophage sub-clusters in IPF. **(F)** Violin plots of Ms4a6c-like signature scores across IPF macrophage sub-clusters. **(G)** Violin plot comparing EMT activity between Ms4a6c-low and Ms4a6c-high macrophages in the IPF cohort. **(H)** Violin plot comparing TGF-β signaling activity between Ms4a6c-low and Ms4a6c-high macrophages in the IPF cohort. **(I)** SCENIC regulon specificity scores (rSS) ranking for fibroblasts (Fib), Ms4a6c+ macrophages (Ms4a6c+Mac), and Ms4a6c− macrophages (Ms4a6c−Mac), with top-ranked transcription factors annotated. **(J)** rSS ranking for Ms4a6c+Mac, highlighting Maf, Cebpa, Bach1, Cebpb, and Rel. **(K)** rSS ranking for Ms4a6c−Mac, highlighting Etv3, Etv6, Irf5, Rel, and Crem. **(L)** Lollipop plot of candidate transcriptional regulators of Ms4a6c ranked by importance score, with Cebpb highlighted.

To ensure these differences were not driven by pseudoreplication, we repeated the comparisons at the sample level, averaging EMT and TGF-β scores per animal or patient. Ms4a6c-high macrophages retained significantly higher activity in the mouse CRP (paired t test, EMT P = 0.037, TGF-β P = 0.001) and IPF (EMT P = 7.0e-04, TGF-β P = 0.021) datasets; in liver cirrhosis (n=5 patients), the TGF-β direction was concordant but did not reach significance (P = 0.088), whereas EMT showed no significant difference (P = 0.548; **Supplementary Figure S10**). These cross-disease analyses suggest that the Ms4a6c+ macrophage phenotype may represent a conserved pro-fibrotic macrophage state across multiple fibrotic conditions, although this inference is based on transcriptomic scoring rather than direct functional assays.

Furthermore, SCENIC regulon analysis identified distinct transcription factor profiles across cell types: fibroblasts were enriched for Gata3, Zfp1, Meis2, Ets1, and Tef (**Figure 5I**), while Ms4a6c+ macrophages exhibited high specificity for Maf, Cebpa, Bach1, Cebpb, and Rel (**Figure 5J**), in contrast to Ms4a6c− macrophages characterized by Etv3, Etv6, Irf5, Rel, and Crem (**Figure 5K**). Among all candidate regulators, Cebpb was supported by motif enrichment (**Figure 5J**, **Supplementary Figure S5**) and the highest regulon specificity score in Ms4a6c+ cells (**Figure 5L**), suggesting it may serve as a candidate upstream transcription factor associated with the Ms4a6c+ macrophage phenotype. However, direct regulatory relationships require validation through ChIP-seq or luciferase reporter assays.

### *In silico* Ms4a6c knockout predicts attenuation of pro-fibrotic signatures and prioritizes MOTS-c as a candidate ligand

Using scTenifoldKnk, a gene regulatory network (GRN) perturbation method, single-gene in silico knockout of Ms4a6c predicted transcriptomic reprogramming (**Figure 6A**), including predicted downregulation of pro-fibrotic signatures and predicted upregulation of epithelial genes Cdh1, Cldn7, and Dstn (**Figures 6B, C**). Pathway enrichment of the predicted changes highlighted tight and adherens junction pathways (**Figures 6D, E**). Although Cdh1 and Cldn7 are classically epithelial genes, Cdh1 (E-cadherin) is intrinsically expressed in the monocyte-macrophage lineage and is an established marker of alternatively activated (M2) macrophages, in which the E-cadherin/catenin complex regulates macrophage activation and fusion (29, 30); their predicted change upon Ms4a6c perturbation is therefore consistent with a macrophage-intrinsic adhesion program rather than epithelial contamination. We emphasize that these are computational predictions of a network-perturbation model rather than experimental knockout results, and they nominate Ms4a6c as a candidate target warranting experimental validation. Structural alignment confirmed conservation between murine Ms4a6c and human MS4A6A (**Supplementary Figures S6A–B**). Virtual screening prioritized four candidates (MOTS-c, Omiganan, Kisspeptin, PKI) based on their docking scores (**Figures 6F–J**, **Supplementary Table 4**). In 100-ns MD simulations, MOTS-c exhibited superior predicted stability, characterized by a low, convergent RMSD (0.6–0.8 nm; **Figure 7A**), rigid RMSF profile (**Figure 7B**), and stable radius of gyration (**Figure 7C**). Unlike Kisspeptin, which showed conformational heterogeneity (**Supplementary Figure S8**), or Omiganan and PKI, which were unstable (**Supplementary Figure S7**, S9), MOTS-c maintained a singular deep energy minimum in FEL analysis (**Figures 7E, F**) and sustained close-range interactions (**Figure 7D**). Thus, MOTS-c was identified as the top in silico screening hit (AutoDock binding energy −5.5 kcal/mol, a moderate-to-weak score), nominated for future experimental validation.

**Figure 6:**
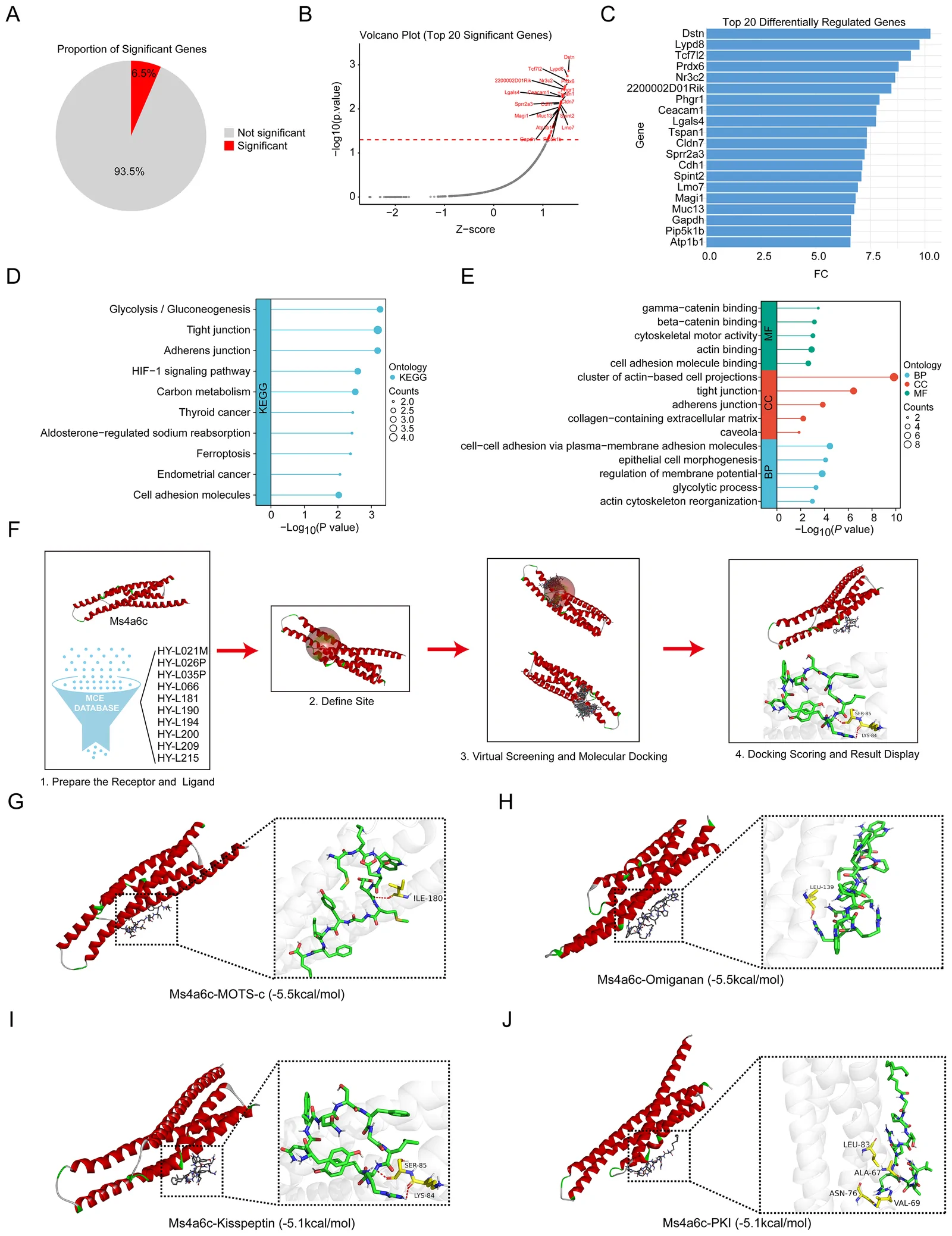
Single-cell virtual knockout simulation of Ms4a6c and screening for potential peptide-based modulators. **(A)** Proportion of altered genes following Ms4a6c virtual knockout. **(B, C)** Volcano plot and bar chart of top 20 DEGs. **(D, E)** KEGG and GO enrichment of DEGs. **(F)** Workflow of structure-based virtual screening. **(G–J)** Molecular docking of Ms4a6c with MOTS-c, Omiganan, Kisspeptin, and PKI. Protein: red ribbons; Ligands: green sticks; Key residues: yellow; Hydrogen bonds: red dashed lines. Binding energies are indicated.

**Figure 7:**
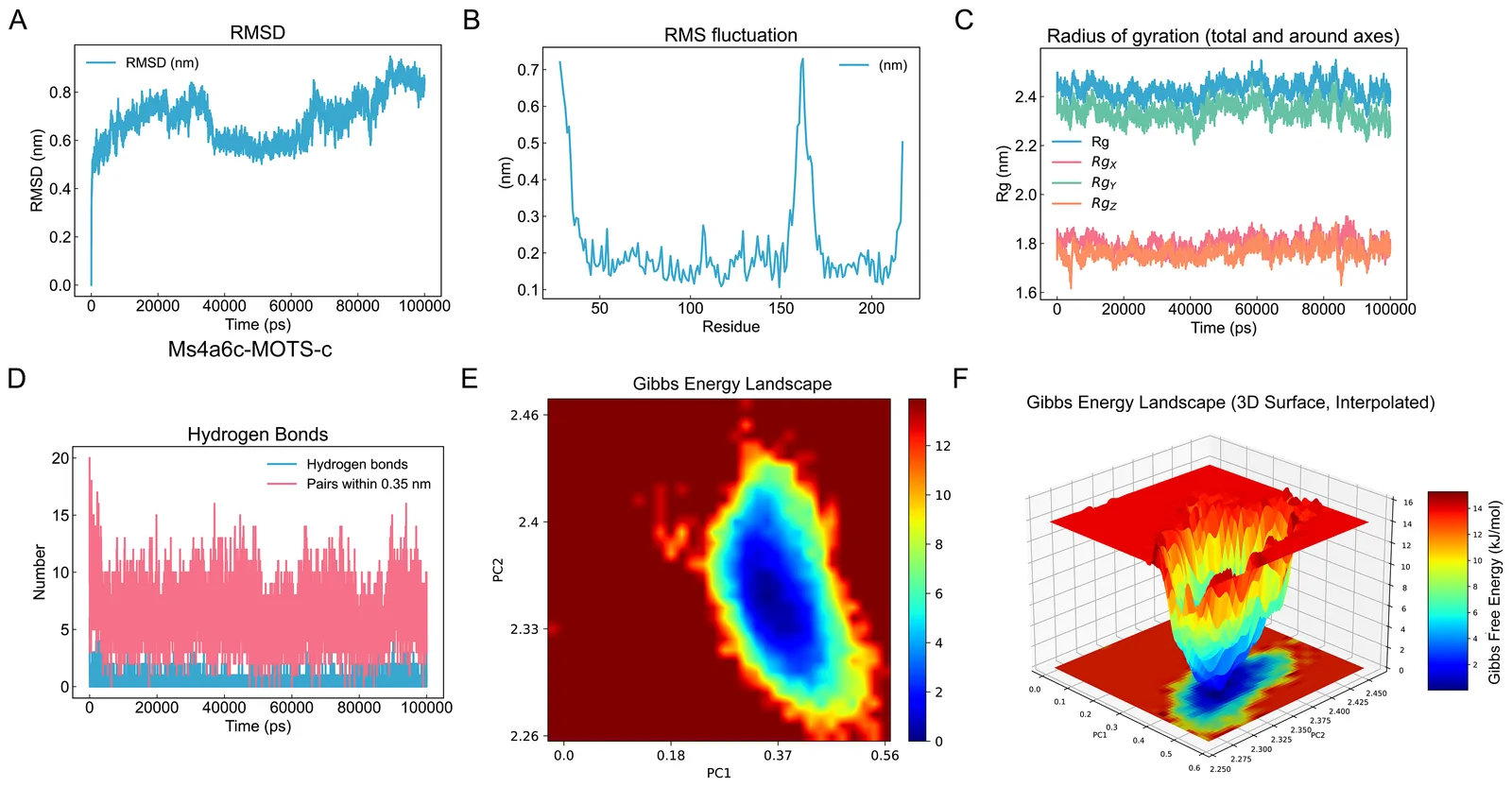
Molecular dynamics simulation identifies MOTS-c as the computationally most stable predicted binding partner for Ms4a6c. **(A)** RMSD plot of the Ms4a6c-MOTS-c complex (20–100 ns). **(B)** RMSF analysis showing localized flexibility (residue ~160). **(C)** Radius of Gyration (Rg) analysis. **(D)** Number of hydrogen bonds (cyan) and contact pairs <0.35 nm (pink). **(E, F)** 2D and 3D Gibbs free energy landscapes showing a single energy minimum.

## Discussion

Chronic radiation proctitis (CRP) poses a substantial clinical challenge, affecting 5-20% of patients undergoing pelvic radiotherapy, yet no pharmacological interventions have been approved by the FDA (31, 32). Utilizing integrative transcriptomic and computational analysis, we have identified Ms4a6c as a macrophage-specific marker associated with pro-fibrotic gene signatures in radiation-induced intestinal fibrosis. Cross-disease computational analysis in liver cirrhosis and idiopathic pulmonary fibrosis single-cell datasets further supported the potentially conserved pro-fibrotic association of Ms4a6c+ macrophages. Furthermore, we have characterized the Cebpb-Ms4a6c-Thbs1-Cd47 axis as a candidate regulatory pathway that merits functional validation. It is important to note that the current study is primarily based on correlative and computational evidence; establishing causal relationships will require genetic loss-of-function experiments, such as conditional knockout or knockdown of Ms4a6c in macrophages, as well as pharmacological pathway blockade in appropriate animal models.

The identification of Ms4a6c as a macrophage-specific biomarker addresses a critical gap in understanding the immune cell populations that contribute to radiation-induced intestinal fibrosis. Single-cell RNA sequencing demonstrated that Ms4a6c expression is exclusive to macrophages. At the subset level, Ms4a6c+ macrophages showed significantly higher EMT and TGF-β signaling scores than Ms4a6c− macrophages (EMT P = 3.66 × 10^–37^, TGF-β P = 1.56 × 10^–49^). The corresponding single-cell correlations were weaker in magnitude (R ≈ 0.16–0.19; **Figures 3N, O**), as expected for a single gene versus an aggregate pathway score. This finding holds significant implications for several reasons. Firstly, Ms4a6c+ macrophages exhibit a hybrid activation state that integrates M2-like characteristics with inflammatory capabilities, such as NF-κB activation. This challenges the conventional M1/M2 dichotomy and aligns with emerging evidence suggesting that tissue macrophages exist along a continuum of activation states (33, 34). Secondly, our findings are consistent with discoveries in various organs, where distinct macrophage subsets expressing Trem2, SPP1, or FABP5 contribute to fibrosis through convergent extracellular matrix remodeling programs (16, 35). This suggests the existence of therapeutically targetable shared mechanisms. Thirdly, although members of the MS4A family are known to mark tissue-resident macrophages (17), the functional role of Ms4a6c in fibrosis has not been previously explored. Our study provides preliminary computational evidence through in silico analysis; the single-gene virtual knockout of Ms4a6c computationally predicted an attenuation of the pro-fibrotic transcriptomic state, concurrently predicting restoration of the expression of epithelial identity markers such as Cdh1 and Cldn7. This suggests that Ms4a6c expression may not merely be a passive marker but could functionally participate in maladaptive tissue remodeling, although direct experimental validation through genetic approaches (e.g., conditional deletion or siRNA-mediated knockdown) is essential to confirm this hypothesis. Consequently, pending further validation through *in vivo* genetic approaches such as conditional deletion, Ms4a6c represents a candidate marker of this pro-fibrotic macrophage subset and a potential target for macrophage-directed intervention, both of which require experimental validation.

The putative mechanistic basis of the pro-fibrotic activity of Ms4a6c+ macrophages was inferred through cell-cell communication analysis, which predicted an enhanced Thbs1-Cd47 signaling pathway between Ms4a6c+ macrophages and fibroblasts. Analysis using CellChat indicated that Ms4a6c+ macrophages exhibited the most extensive predicted intercellular communication (43 interactions compared to 7 interactions for fibroblasts), with Thbs1-Cd47 demonstrating the highest communication probability. Multiplex immunofluorescence confirmed the spatial co-localization of Ms4a6c, Thbs1, and Cd47 within CRP tissues. However, co-localization alone does not establish a functional signaling relationship. To establish causality, functional validation through pathway blockade is necessary. Approaches such as employing anti-Thbs1 antibodies, CD47 antagonists, or genetic deletion within our model would directly assess whether disrupting this axis mitigates fibrosis. The computationally proposed pathway is mechanistically plausible: THBS1 activates latent TGF-β and interacts with CD47 or integrin receptors to facilitate fibroblast adhesion, migration, and extracellular matrix production (36, 37). Previous studies demonstrating that THBS1 blockade alleviates fibrosis in the liver, kidney, and lung further support its potential as a therapeutic target (38–40). Alternative receptors for Thbs1, such as Sdc4, Sdc1, and Cd36, imply potential functional redundancy, suggesting that combinatorial targeting strategies may be required.

A key component of this study is the cross-disease analysis of Ms4a6c+ macrophages using independent single-cell datasets from liver cirrhosis (GSE136103) and idiopathic pulmonary fibrosis (GSE135893). In both conditions, macrophage subsets with high Ms4a6c-like signature scores exhibited significantly elevated EMT and TGF-β signaling activity compared to their Ms4a6c-low counterparts, supporting the hypothesis that Ms4a6c+ macrophages represent a conserved pro-fibrotic effector population rather than a tissue-specific phenomenon. It should be noted that this cross-disease comparison relies on computational signature scoring rather than direct functional assays and therefore represents associative rather than causal evidence. Furthermore, SCENIC regulon analysis identified Cebpb as a candidate upstream transcriptional regulator of the Ms4a6c+ macrophage phenotype. The biological plausibility of this finding is supported by the established role of C/EBPβ in alternative macrophage activation and pathological fibrosis across various organs (41–43). Ms4a6c+ macrophages co-express several pro-fibrotic transcription factors, including Cebpb, Maf, Bach1, and Rel, suggesting coordinated transcriptional regulation. However, direct regulatory relationships need to be validated through ChIP-seq, luciferase reporter assays, and Cebpb knockdown/knockout experiments. If validated, targeting Cebpb may prevent pathological macrophage polarization upstream of effector pathways.

Our structure-based virtual screening constitutes an initial computational exploration of potential therapeutics directed against Ms4a6c. Among the screened compounds, the mitochondrial-derived peptide MOTS-c was identified as the top-ranking in silico screening hit predicted to interact with Ms4a6c, with a moderate-to-weak docking score (−5.5 kcal/mol) and relative stability in molecular dynamics simulations. We note, however, that MOTS-c is principally characterized as an intracellular peptide acting through AMPK and Nrf2 signaling, so a direct physical interaction with a tetraspanin-family membrane protein such as Ms4a6c remains mechanistically unproven and warrants direct binding validation. More broadly, it is crucial to recognize significant limitations: these findings are exclusively based on computational modeling without experimental validation. Even if the predicted interaction between MOTS-c and Ms4a6c is biologically valid, several critical questions remain unanswered, including its capacity for functional modulation, its impact on macrophage polarization, its efficacy *in vivo*, and its potential interference with radiotherapy. Moreover, because Ms4a6c is a multi-pass membrane protein, our molecular dynamics simulations were performed in an aqueous environment without an explicit lipid bilayer, which does not fully recapitulate the native membrane context and may affect the predicted binding stability; membrane-embedded simulations will be needed to refine these predictions. Rigorous experimental validation through direct binding assays, such as surface plasmon resonance (SPR) and isothermal titration calorimetry (ITC), along with cellular functional assays and *in vivo* testing, will be essential to confirm these predictions. Nonetheless, the established role of MOTS-c in metabolic immune regulation (44), and its recently identified efficacy in mitigating radiation-induced tissue fibrosis via the Nrf2 signaling pathway (45), render it a compelling candidate for further investigation.

Several limitations warrant emphasis. Firstly, species-specific differences may impede direct translation; thus, validating Ms4a6c expression and macrophage states in human specimens will be crucial. Secondly, our single-cell analysis was restricted to a single, chronic-phase timepoint (8 weeks). This timepoint was chosen deliberately to capture the established fibrotic niche, consistent with prior mouse models of radiation proctitis in which week 8 post-irradiation defines the chronic stage that recapitulates human chronic RP (24), and corresponds to the endpoint used in the localized pelvic-irradiation model that identified PDGF-C as a therapeutic target for radiation proctopathy (23). Nonetheless, a single chronic-phase snapshot cannot distinguish whether Ms4a6c+ macrophages contribute to the onset of fibrosis or associate with already-established fibrotic tissue. Longitudinal single-cell profiling spanning the acute and subacute phases through the chronic phase will be required to resolve these temporal dynamics and to define therapeutic windows. In addition, although we examined the Ms4a6c+ macrophage phenotype in liver cirrhosis and IPF datasets, the cross-disease analysis relies on computational scoring rather than direct functional assays. Crucially, our mechanistic conclusions are primarily based on correlative evidence. While our integrative transcriptomic and computational analysis reveals significant associations among Ms4a6c+ macrophages, Cebpb regulation, Thbs1-Cd47 signaling, and fibrosis, causality has yet to be established. Most critically, the absence of genetic loss-of-function experiments (e.g., Ms4a6c conditional knockout mice, macrophage-specific Cebpb deletion, or siRNA/shRNA-mediated knockdown in primary macrophages) and pharmacological intervention studies (e.g., anti-Thbs1 or anti-CD47 antibody treatment in the CRP model) means that all proposed mechanistic relationships remain hypothetical until experimentally confirmed. Future research should prioritize: (1) validation through loss- and gain-of-function studies in Ms4a6c-deficient or overexpression models; (2) pathway inhibition using anti-Thbs1/CD47 antibodies; (3) experimental validation of the predicted Cebpb-Ms4a6c regulatory relationship via ChIP-seq and luciferase reporter assays; and (4) experimental verification of the predicted MOTS-c/Ms4a6c interaction.

## Conclusions

In summary, this study highlights Ms4a6c+ macrophages as candidate effectors in the maladaptive remodeling characteristic of chronic radiation proctitis. By integrating transcriptomic and computational modeling with cross-disease analysis in liver cirrhosis and idiopathic pulmonary fibrosis, we identify the Cebpb-Ms4a6c-Thbs1-Cd47 axis as a candidate pro-fibrotic signaling cascade potentially conserved across organ systems. Furthermore, the identification of MOTS-c as a computationally predicted candidate modulator provides a preliminary lead for future pharmacological investigation. These findings begin to unravel the cellular heterogeneity of radiation injury and offer a computational framework toward developing targeted therapies applicable across multiple fibrotic diseases. Future work incorporating genetic and pharmacological functional validation will be essential to translate these computational findings into therapeutic strategies.

## Data Availability

The datasets presented in this study can be found in online repositories. The names of the repository/repositories and accession number(s) can be found below: Genome Sequence Archive (GSA), National Genomics Data Center, China National Center for Bioinformation (https://ngdc.cncb.ac.cn/gsa), BioProject PRJCA057809, GSA accession CRA044988 (https://ngdc.cncb.ac.cn/gsa/browse/CRA044988). The publicly available datasets analyzed in this study are available in the Gene Expression Omnibus under accession numbers GSE136103, GSE135893 and GSE159112.
