# Supplementary material for: Integrative transcriptomic and computational analysis associates Ms4a6c+ macrophages with fibrosis in radiation proctitis and computationally nominates MOTS-c as a candidate modulator
Source: Front Immunol. 2026 Oct 14;17:1907084. doi: 10.3389/fimmu.2026.1907084 (PMC13713458; doi:10.3389/fimmu.2026.1907084)
Supplement: Supplementary file 1 [file DataSheet1.pdf]

Supplementary Figures and tables

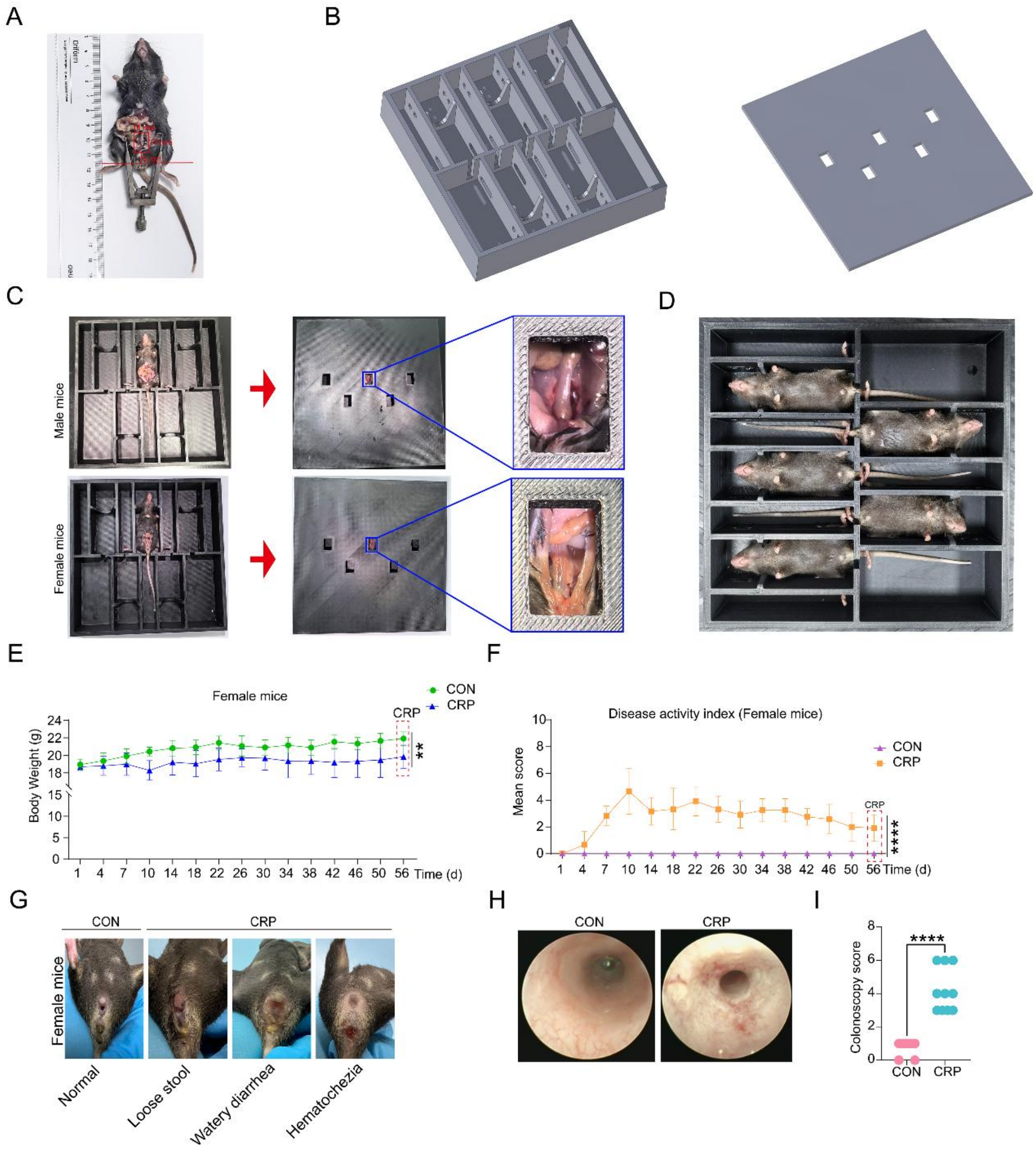

Supplementary Fig. 1. Design of Custom 3D-Printed Irradiation Device and Validation of CRP Model in Female Mice

(A) An anesthetized mouse is positioned within the irradiation mold. (B) A SolidWorks 3D design illustrates a positioning device accommodating five mice, featuring a lead shielding plate with apertures measuring 10 mm × 15 mm. (C) Views of the assembled mold and lead shielding plate are presented. (D) The complete irradiation setup includes five mice. (E-F)

Measurements of body weight and Disease Activity Index (DAI) are reported for female mice, with a sample size of n=10 per group. (G) Representative gross pathological images illustrating diarrhea and hematochezia in CRP female mice. (H-I) Colonoscopic images and corresponding quantitative scores are provided for female mice. Data are expressed as mean ± SEM.

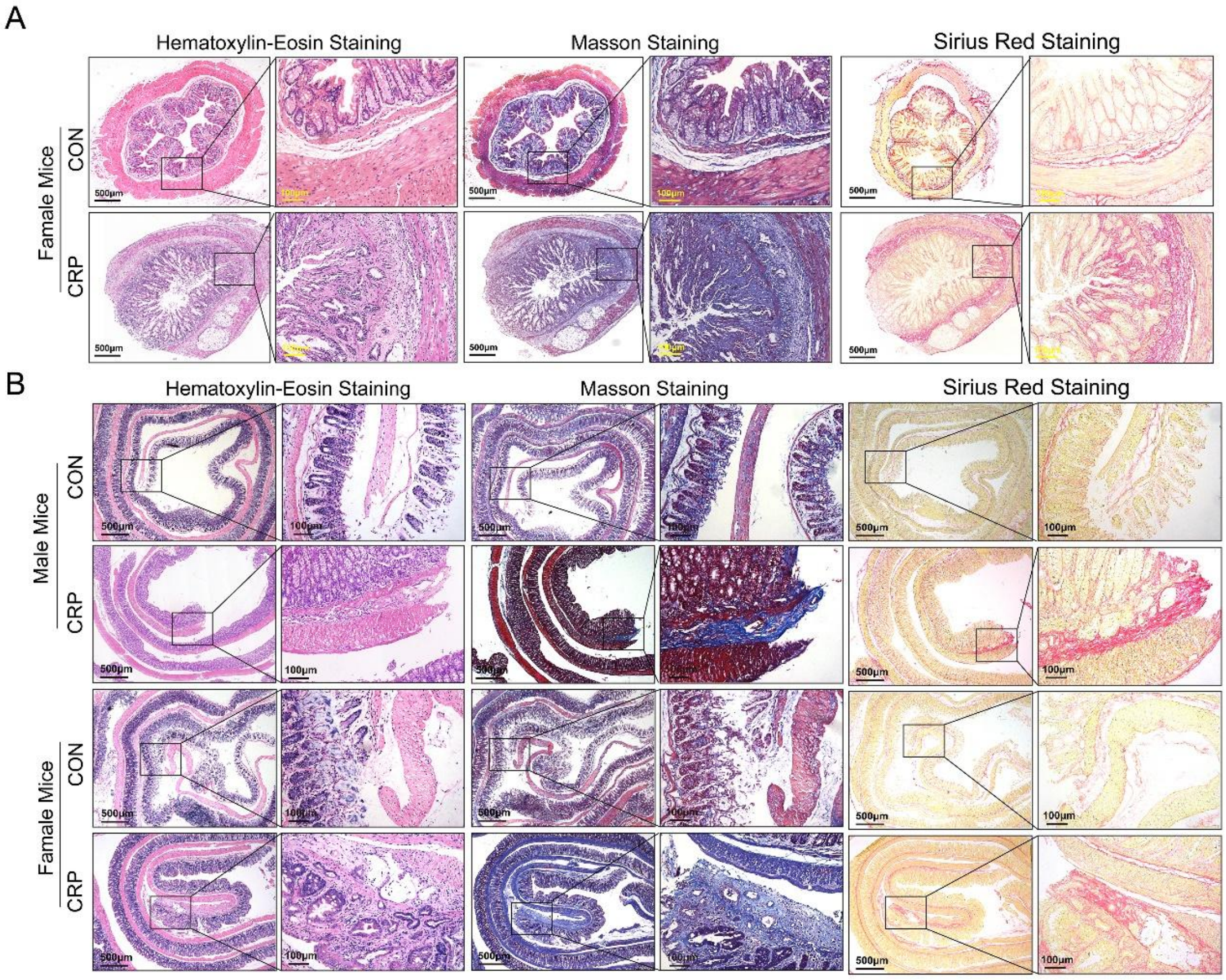

Supplementary Fig. 2. Regional Specificity of Radiation-Induced Fibrosis Demonstrating Precise Targeting of the Distal Rectum

(A) Cross-sectional histological images from female mice display isolated intestinal segments stained with Hematoxylin and Eosin (H&E) on the left, Masson's trichrome in the center (highlighting collagen in blue), and Sirius Red on the right (highlighting collagen in red). The scale bars represent 500 μm and 100 μm, respectively. (B) Longitudinal sections of complete

colorectal segments from both male and female mice are presented with three distinct staining modalities.

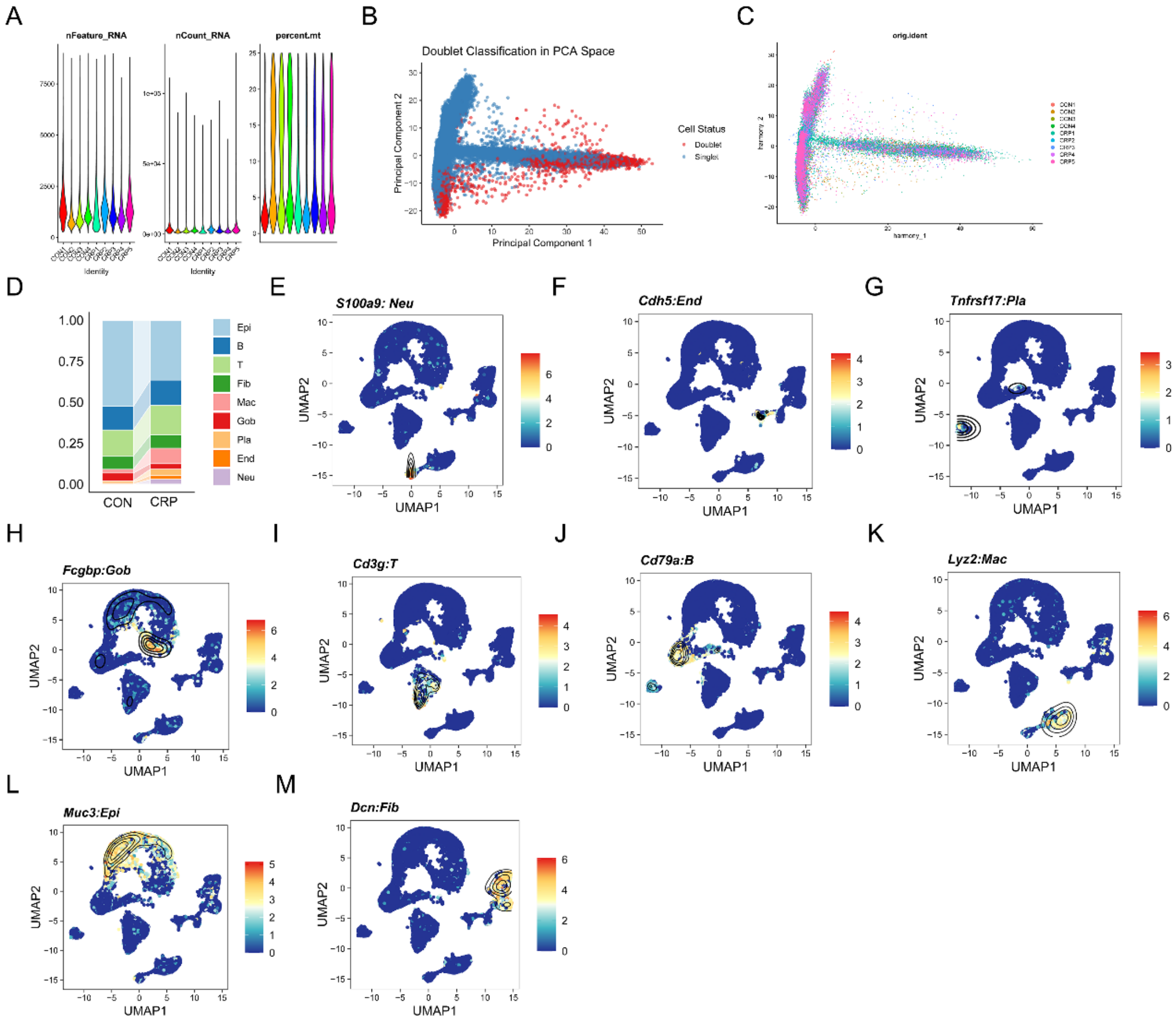

Supplementary Fig. 3. Quality Control Metrics and Cell Type Annotation for Single-Cell RNA Sequencing Analysis

(A) Violin plots illustrate quality control metrics, including nFeature_RNA (left), nCount_RNA (middle), and percent.mt (right). (B) Doublet classification is depicted in PCA space using DoubletFinder, with singlets represented in blue and doublets in red. (C) UMAP projection is colored according to sample origin, ranging from CON1 to CON4 and CRP1 to CRP5. (D) A bar plot compares cell type proportions between control and CRP samples. (E-M) UMAP plots

demonstrate the expression of canonical marker genes.

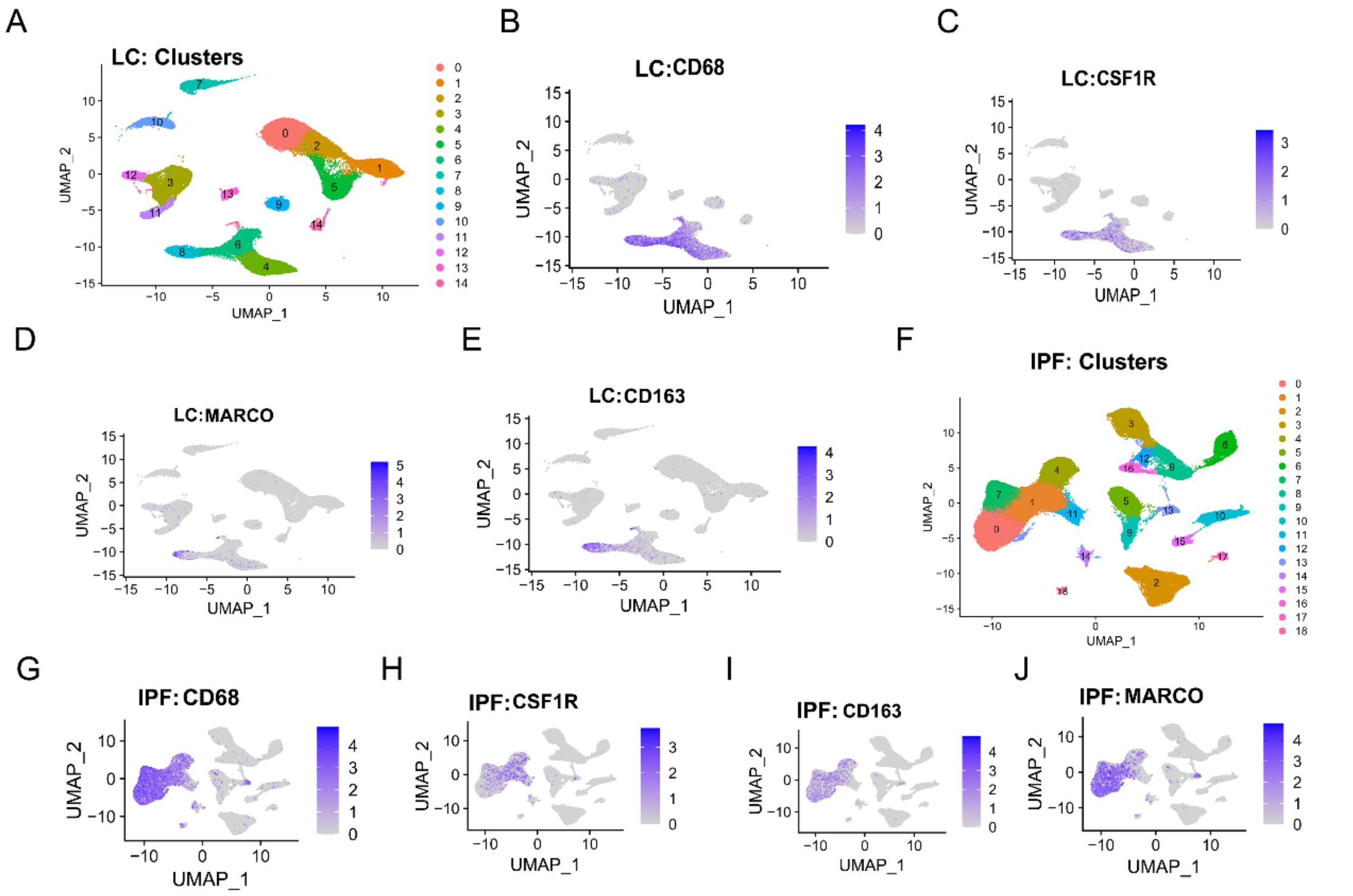

Supplementary Fig. 4. Macrophage sub-cluster annotation and marker expression in liver cirrhosis and idiopathic pulmonary fibrosis datasets. (A) UMAP visualization and macrophage cluster identification in the LC dataset (GSE136103). (B-E) Expression of macrophage markers CD68, CSF1R, CD163, and MARCO in the LC dataset. (F) UMAP visualization and macrophage cluster identification in the IPF dataset (GSE135893). (G-J) Expression of macrophage markers CD68, CSF1R, CD163, and MARCO in the IPF dataset.

A

Display 10 profiles | Filter:

Matrix ID	Name	Score	Relative score	Sequence ID	Start	End	Strand	Predicted sequence
MA0466.1	MA0466.1.CEBPB	10.358004	0.9285134	NC_000011.10:c60186666-60171597	1349	1359	+	TGTTACACAAC
MA0466.1	MA0466.1.CEBPB	10.169044	0.92653275	NC_000011.10:c60186666-60171597	944	954	-	GCTTGCTCAAT
MA0466.1	MA0466.1.CEBPB	9.742508	0.9220617	NC_000011.10:c60186666-60171597	1461	1471	-	AATTTCTCAAC
MA0466.1	MA0466.1.CEBPB	6.2449107	0.8853994	NC_000011.10:c60186666-60171597	603	613	-	AATTGCCCCAG

B

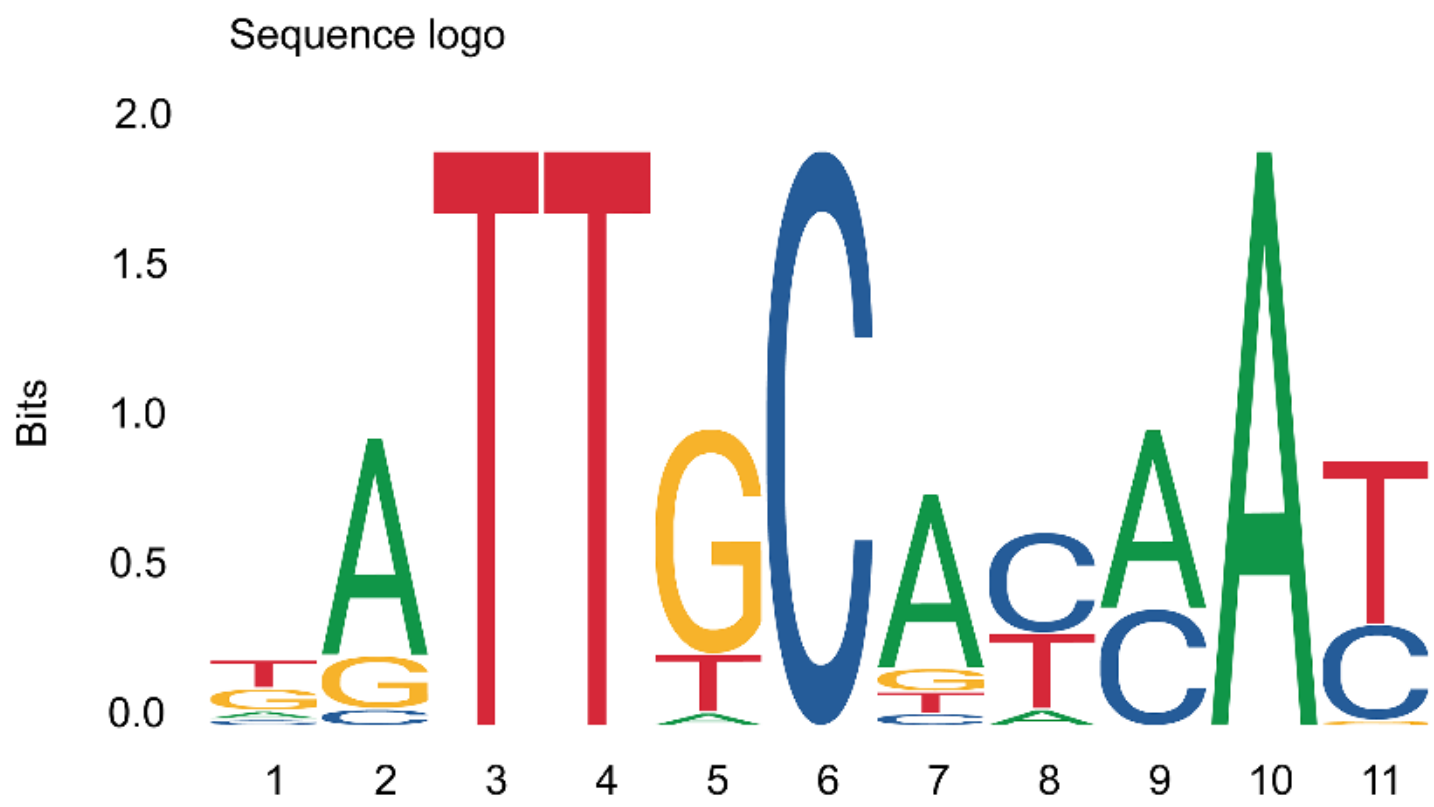

Supplementary Fig. 5. Motif enrichment analysis predicts Cebpb as a transcriptional regulator of Ms4a6c. (A) Predicted transcription factor binding sites within the Ms4a6c promoter region are identified. The table enumerates specific high-scoring binding sequences identified by JASPAR, with relative profile scores exceeding 6.0, thereby supporting the regulatory potential of Cebpb on Ms4a6c expression. **(B)** The sequence logo of the Cebpb binding motif (JASPAR Matrix ID: MA0466.1) illustrates the high conservation of the binding sequence.

A

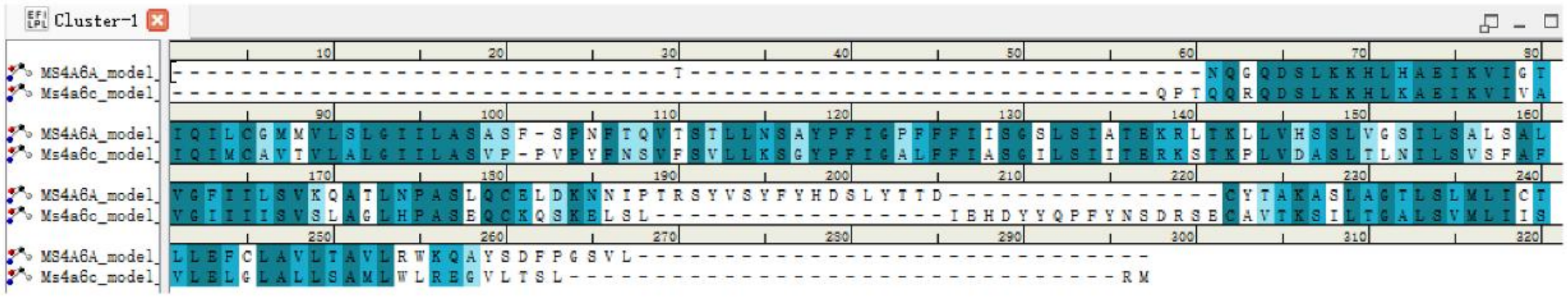

B

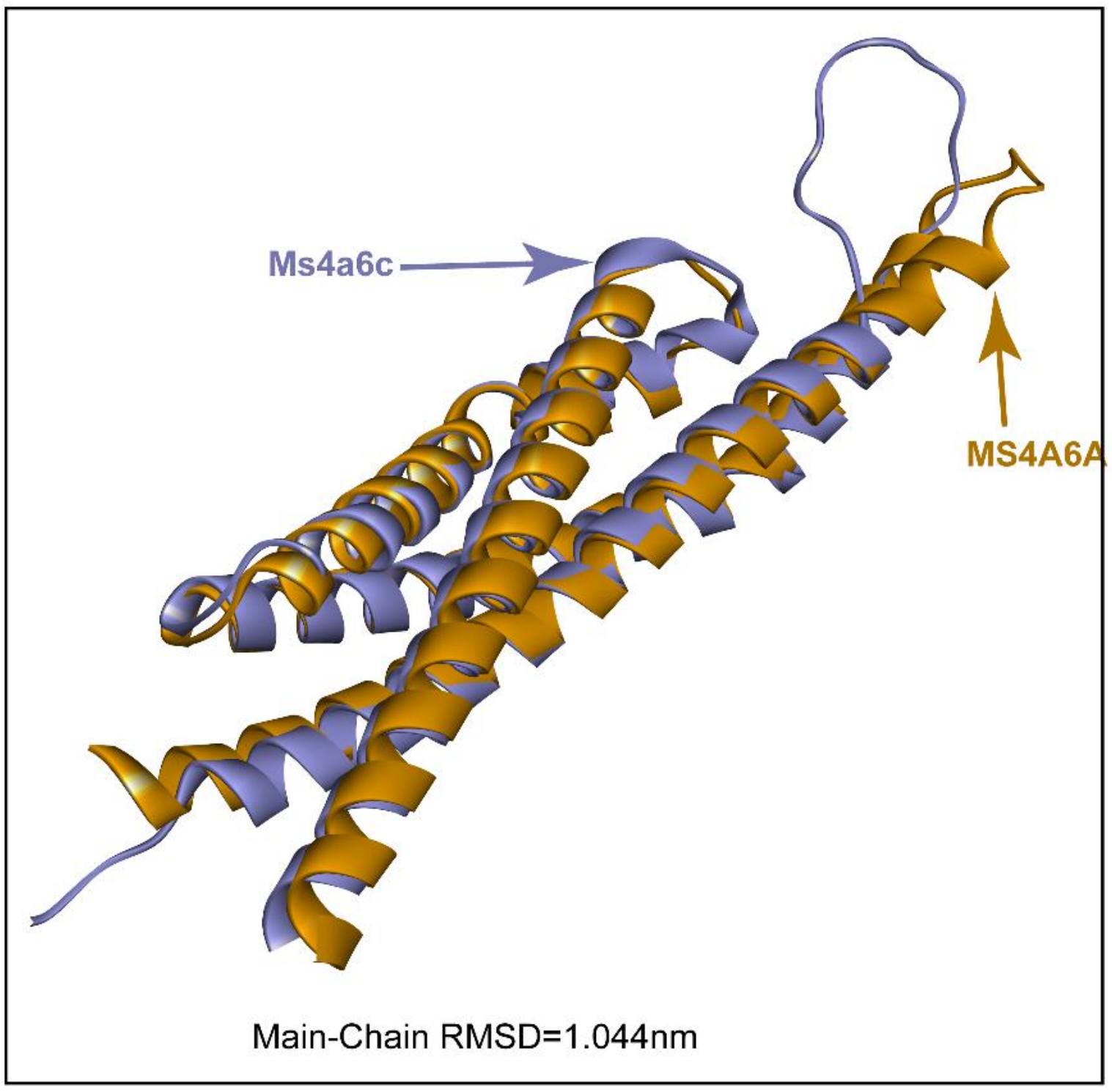

Supplementary Fig. 6. Structural conservation analysis between mouse Ms4a6c and human MS4A6A. (A) The sequence alignment of the mouse Ms4a6c protein with its human ortholog, MS4A6A, highlights identical and highly conserved amino acid residues in teal, underscoring sequence homology within critical functional domains. **(B)** A three-dimensional structural superposition of the mouse Ms4a6c (depicted as a purple ribbon) and human MS4A6A (depicted as an orange ribbon) protein models reveals a consistent transmembrane helical architecture. This alignment yields a Main-Chain Root Mean Square Deviation (RMSD) of 1.044 nm, indicating substantial structural similarity and the potential for conserved ligand-binding mechanisms across species.

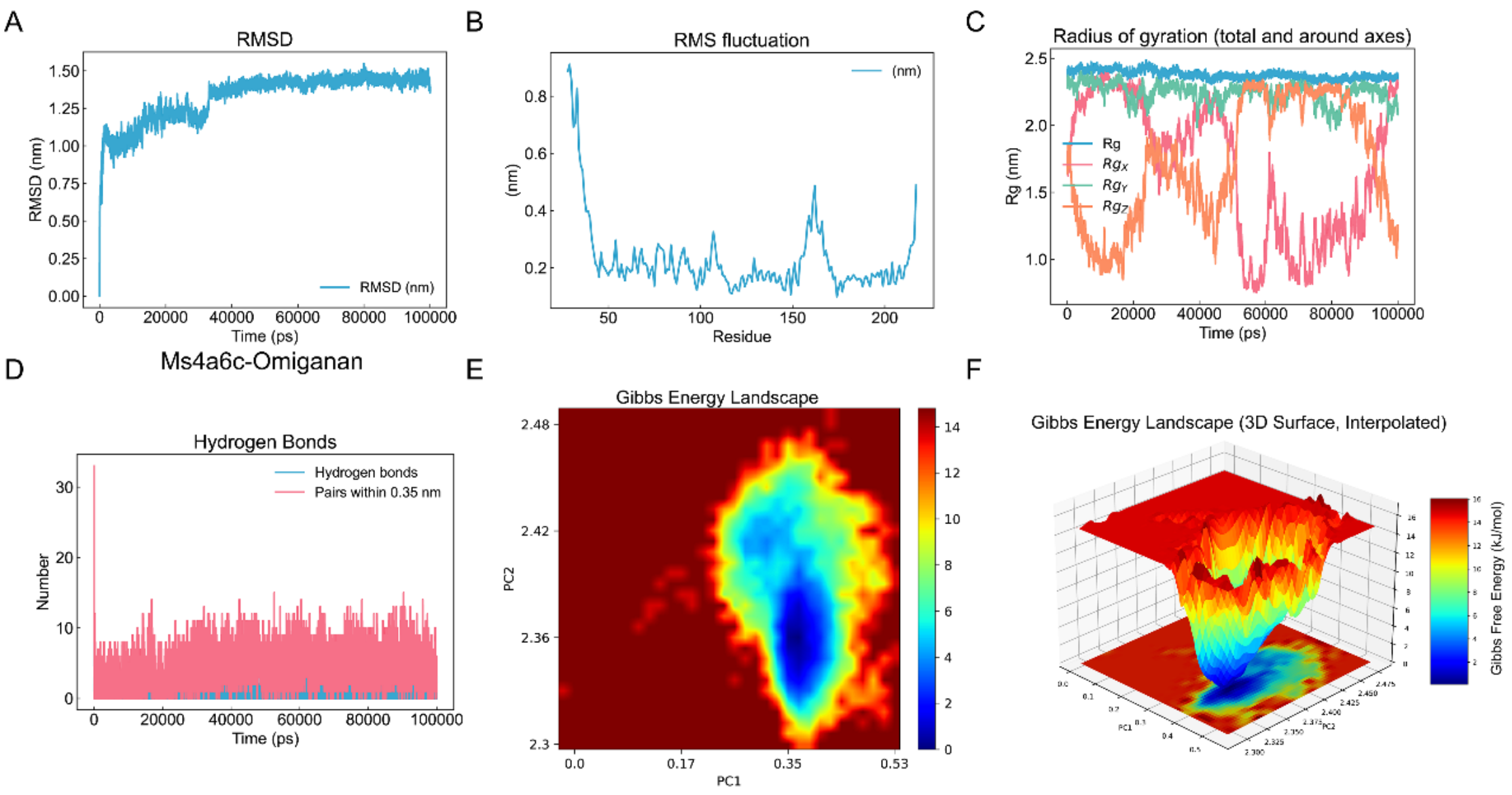

Supplementary Fig.7. Molecular dynamics simulation of Ms4a6c-Omiganan complex. (A) The RMSD analysis indicates a continuous upward trend, exceeding 1.4 nm, which suggests structural instability. **(B)** The Root Mean Square Fluctuation (RMSF) plot. **(C)** The radius of gyration analysis demonstrates significant fluctuations in structural compactness. **(D)** Interaction analysis. **(E-F)** The Gibbs free energy landscapes exhibit a broad, dispersed energy basin without a defined minimum, implying weak binding stability.

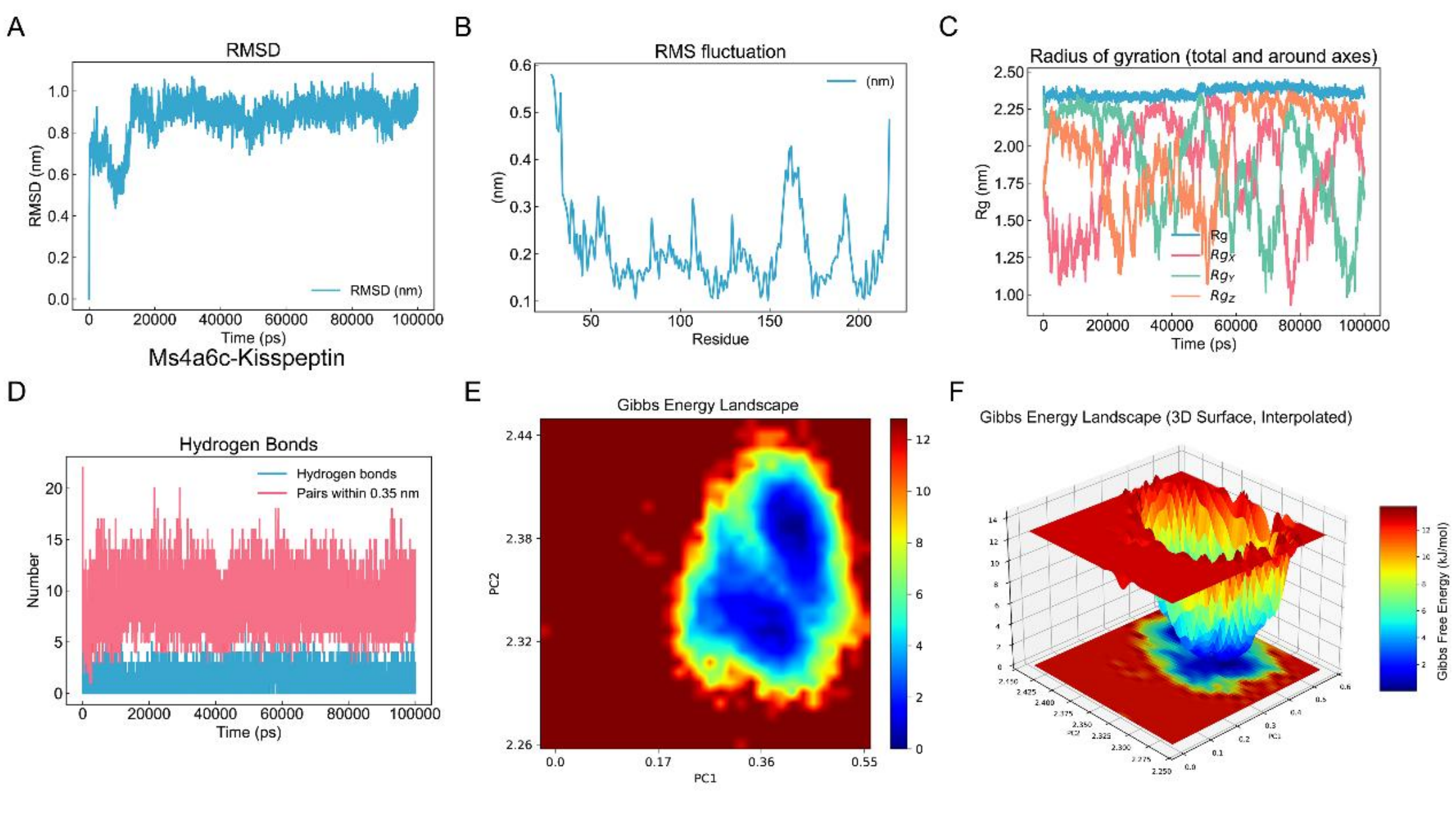

Supplementary Fig. 8. Molecular dynamics simulation of Ms4a6c-Kisspeptin complex. (A)

The RMSD trajectory stabilizes within the range of 0.8–1.0 nm, indicating moderate stability but exhibiting greater deviation in comparison to MOTS-c. **(B)** RMSF analysis. **(C)** The radius of gyration suggests a compact structural configuration. **(D)** Interaction analysis reveals fluctuating contact pairs. **(E-F)** The Gibbs free energy landscapes demonstrate a bimodal character with two distinct energy minima regions, suggesting that the complex oscillates between two conformational states rather than maintaining a single stable conformation.

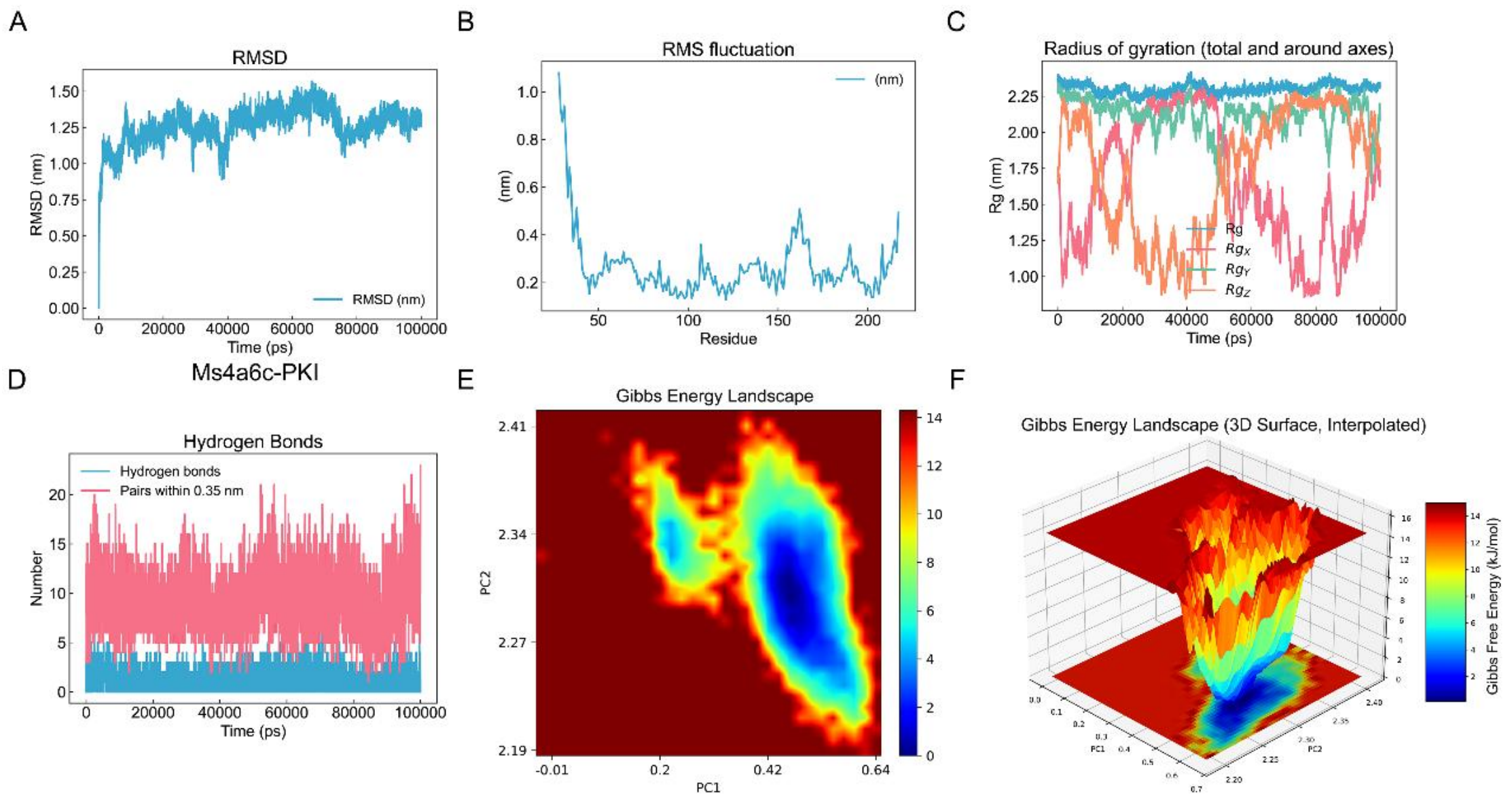

Supplementary Fig. 9. Molecular dynamics simulation of Ms4a6c-PKI complex. (A) The RMSD plot indicates a high deviation (~1.25 nm). **(B)** RMSF analysis. **(C)** Radius of gyration. **(D)** Hydrogen bond analysis. **(E-F)** The Gibbs free energy landscapes exhibit multiple separated energy minima, indicating conformational heterogeneity and unstable binding.

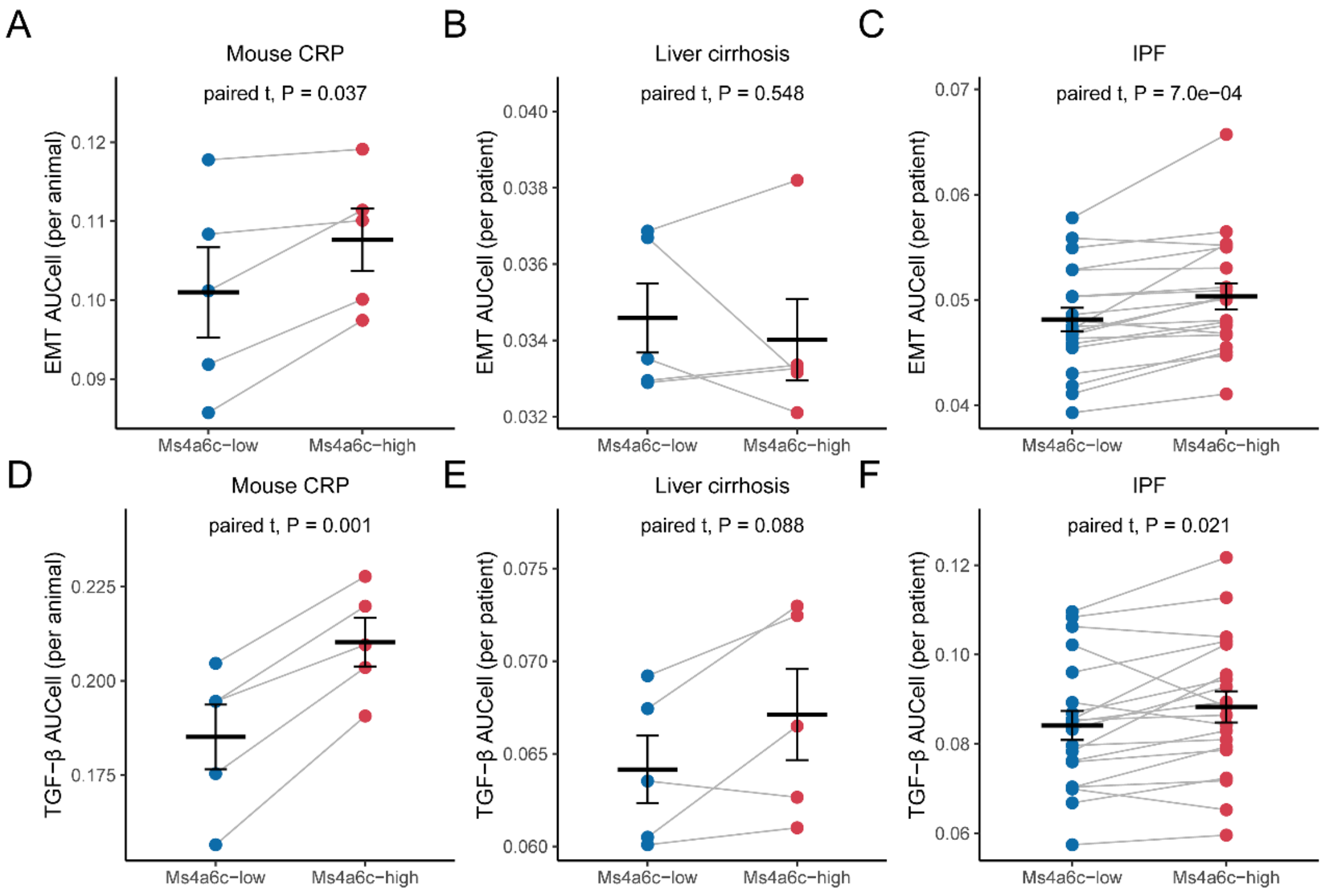

Supplementary Fig. 10. Sample-level (pseudobulk) validation of pro-fibrotic activity in Ms4a6c-high macrophages. Cell-level comparisons were repeated at the sample level by averaging EMT and TGF-β AUCell scores per animal or patient (Ms4a6c-high vs Ms4a6c-low, top and bottom 20% within each sample). (A-C) EMT activity in mouse CRP (n=5), liver cirrhosis (n=5), and IPF (n=20). (D-F) TGF-β activity in the same datasets. Each line connects one sample; group mean ± SEM shown; P from paired t test.

Supplementary Table 1. Scoring system for disease activity index based on body weight loss, Diarrhea, and Hematochezia.

Item	Score[a]
body weight loss	0: None
	1: 1%-5%
	2: 5%-10%
	3: 10%-20%
	4: >20%
Diarrhea	0: normal
	2: loose stools
	4: watery diarrhea
Hematochezia	0: No bleeding
	2: Slight bleeding
	4: Gross bleeding

[a]The total score of three items ranges from 0 to 12. A higher score indicates aggravated radiation damage.

Supplementary Table 2. Mice endoscopic scoring system.

Item	Score[a]
Congested mucosa	0: None
	1: Localized mucosal redness and swelling
	2: Diffuse, non-confluent mucosal redness and swelling
	3: Diffuse and confluent mucosal redness and swelling
Ulceration	0: None
	1: Present or absent with superficial ulcers less than 1 cm^2
	2: Area greater than 1 cm^2
	3: Deep ulcers
	4: Deep ulcers resulting in fistulas or perforation
Telangiectasia	0: None
	1: Single capillary dilation
	2: Multiple, non-confluent capillary dilations
	3: Multiple, confluent capillary dilations.
Stricture	0: None
	1: Greater than 2/3 of the original intestinal lumen diameter
	2: 1/3 to 2/3 of the original intestinal lumen diameter
	3: Less than 1/3 of the original intestinal lumen diameter
	4: Complete obstruction
Necrosis	0: None
	1: Present

[a]The total score of five items ranges from 0 to 15. A higher score indicates aggravated radiation damage.

Supplementary Table 3. Histopathological scoring system for radiation-induced bowel injury.

Item	Score[a]
Mucosal erosions/ulcerations	0: None
	1: Superficial erosions
	2: Ulcerations
Nuclear abnormalities of epithelial cells (Surface & Crypt)	0: None
	1: Hyperchromatic, enlarged nuclei with irregular contours; atypical mitotic figures
Crypt disarrangement	0: None
	1: Crypt disarray
	2: Crypt distortion with branching or shortening
Mucosal inflammatory infiltrate	0: None
	1: Mild increase in cellularity of the lamina propria
	2: Moderate increase in lymphocytes and plasma cells; eosinophilic crypt abscess
	3: Marked increase in lymphocytes and plasma cells with aggregation as ulcerative colitis
Mucosal edema	0: None
	1: Broadened mucosa with scattered cellularity
Fibrosis of the lamina propria	0: None
	1: Increase in hyalinized collagen fibers
Fibrosis of submucosa	0: None
	1: Mild increase in broadened and hyalinized collagen fibers
	2: Moderate increase in dense collagen fibers; increased submucosal thickness
	3: Marked fibers including muscularis
Sclerosis of submucosal vessels	0: None
	1: Fibromuscular thickening of the intima
	2: Moderate thickening of the intima with luminal stenosis
	3: Extreme sclerosis with marked stenosis or occlusion
Lymph congestion	0: None
	1: Dilated lymph vessels or cystic collections of lymph

[a]The total score of nine items ranges from 0 to 17. A higher score indicates aggravated radiation damage.

Supplementary Table 4. LibDock scores and molecular information of the top 20 predicted Ms4a6c ligands identified via virtual screening.

Name	CAS	Catalog_NO	LibDockScore
MOTS-c (mouse)		HY-P10769	228.113
Omiganan	204248-78-2	HY-105048	225.494
Kisspeptin-10 (mouse, rat)	478507-53-8	HY-P1197	225.37
PKI	201422-03-9	HY-P1291	216.398
Chemerin-9 (149-157) (TFA)		HY-P1844A	202.342
KT-333	2502186-79-8	HY-156730	199.273
KT-333 (ammonium)	2839758-34-6	HY-156730A	198.68
5-FAM-GpYLPQTV-NH2	769166-98-5	HY-P10202	197.509
TAT (48-57)	253141-50-3	HY-P1575	196.251
Acetyl hexapeptide-1 (acetate)	2828433-36-7	HY-P5220A	189.367
MSH	581-05-5	HY-P0252	188.627
TMX-201	1149339-78-5	HY-150158	184.781
Cetrorelix (Acetate)	145672-81-7	HY-P0009A	184.02
Multi-Leu peptide (triacetate)		HY-P10422A	181.924
Red pigment-concentrating hormone	37933-92-9	HY-P3824	181.602
Angiopeptin (TFA)	2478421-60-0	HY-P2090A	180.402
BIM-26226	136207-23-3	HY-P0039	179.045
WYRGRL	1021155-39-4	HY-P10739	178.28

Prolyl endopeptidase inhibitor 2	2362592-81-0	HY-162452	177.568
Pentixafor	1341207-62-2	HY-153549	177.083

Supplementary Table 5. Number of cells per macrophage cluster and Ms4a6c-defined subset in the single-cell datasets analyzed in this study.

Dataset	Cluster / subset	Number of cells
CRP scRNA-seq (this study)	Ms4a6c+ macrophages	2,028
	Ms4a6c− macrophages	2,028
Liver cirrhosis (GSE136103)	C0	2,027
	C1	3,260
	C2	1,926
	C3	1,780
	C5	726
	C6	501
	Total macrophages	10,220
	Ms4a6c-high	852
	Ms4a6c-low	852
Idiopathic pulmonary fibrosis (GSE135893)	C0	9,532
	C1	6,689
	C2	6,159
	C3	5,977
	C4	4,503
	C5	3,356
	C6	2,524
	C7	1,493
	C8	407
	Total macrophages	40,640
	Ms4a6c-high	5,559
	Ms4a6c-low	5,559

Supplementary Table 6. Complete 52-gene Ms4a6c-like macrophage signature used for cross-disease scoring (AddModuleScore).

APOE	C1QA	C1QB	C1QC	C5AR1	CCL2
CCL6	CCL7	CCL8	CCL9	CCL12	CD47
CEBPB	CLEC4A1	CSF1R	CTSB	CTSS	DUSP1
EGR1	FCER1G	FCGR1	FCGR4	FOLR2	GAS6
GRN	IFI27L2A	IFITM2	IFITM3	ITM2B	LST1
LY6A	LY6E	LYZ2	MAF	MAFB	MARCKSL1
MRC1	MS4A4C	MS4A6A	MS4A6B	MS4A6D	MS4A7
PDE7B	PF4	PLA2G7	PLAC8	PLTP	SELENOP
THBS1	TMEM176B	TYROBP	WFDC17		
